# Human iPSC and CRISPR targeted gene knock-in strategy for studying the somatic TIE2^L914F^ mutation in endothelial cells

**DOI:** 10.1007/s10456-024-09925-9

**Published:** 2024-05-21

**Authors:** Bojana Lazovic, Hoang-Tuan Nguyen, Mohammadhassan Ansarizadeh, Leif Wigge, Franziska Kohl, Songyuan Li, Miguel Carracedo, Jere Kettunen, Luc Krimpenfort, Ramy Elgendy, Kati Richter, Laknee De Silva, Bilada Bilican, Prateek Singh, Pratik Saxena, Lars Jakobsson, Xuechong Hong, Lauri Eklund, Ryan Hicks

**Affiliations:** 1 BioPharmaceuticals R&D Cell Therapy Department, Research and Early Development, Cardiovascular, Renal, and Metabolism (CVRM), BioPharmaceuticals R&D, AstraZeneca, Gothenburg, Sweden; 2 Translational Genomics, Discovery Sciences, BioPharmaceuticals R&D, AstraZeneca, Gothenburg, Sweden; 3 Oulu Center for Cell‐Matrix Research, Biocenter Oulu and Faculty of Biochemistry and Molecular Medicine, University of Oulu, Oulu, Finland; 4 Finnadvance Ltd., Oulu, Finland; 5 Data Sciences and Quantitative Biology, Discovery Sciences, BioPharmaceuticals R&D, AstraZeneca, Gothenburg, Sweden; 6 Bioscience Renal, Research and Early Development, Cardiovascular, Renal and Metabolism (CVRM), BioPharmaceuticals R&D, AstraZeneca, Gothenburg, Sweden; 7 Department of Medical Biochemistry and Biophysics, Karolinska Institutet, Stockholm, Sweden; 8 School of Cardiovascular and Metabolic Medicine & Sciences, King’s College London, London, UK

**Keywords:** iPSC, Venous malformations, Gene editing, Endothelial cells, TIE2

## Abstract

Induced pluripotent stem cell (iPSC) derived endothelial cells (iECs) have emerged as a promising tool for studying vascular biology and providing a platform for modelling various vascular diseases, including those with genetic origins. Currently, primary ECs are the main source for disease modelling in this field. However, they are difficult to edit and have a limited lifespan. To study the effects of targeted mutations on an endogenous level, we generated and characterized an iPSC derived model for venous malformations (VMs). CRISPR-Cas9 technology was used to generate a novel human iPSC line with an amino acid substitution L914F in the TIE2 receptor, known to cause VMs. This enabled us to study the differential effects of VM causative mutations in iECs in multiple in vitro models and assess their ability to form vessels in vivo. The analysis of TIE2 expression levels in TIE2^L914F^ iECs showed a significantly lower expression of TIE2 on mRNA and protein level, which has not been observed before due to a lack of models with endogenous edited TIE2^L914F^ and sparse patient data. Interestingly, the TIE2 pathway was still significantly upregulated and TIE2 showed high levels of phosphorylation. TIE2^L914F^ iECs exhibited dysregulated angiogenesis markers and upregulated migration capability, while proliferation was not affected. Under shear stress TIE2^L914F^ iECs showed reduced alignment in the flow direction and a larger cell area than TIE2^WT^ iECs. In summary, we developed a novel TIE2^L914F^ iPSC-derived iEC model and characterized it in multiple in vitro models. The model can be used in future work for drug screening for novel treatments for VMs.

**Supplementary Information:**

The online version contains supplementary material available at 10.1007/s10456-024-09925-9.

## Introduction

Primary endothelial cells (ECs) derived from healthy human donors are commonly used in vascular models to study the physiological function and pathological changes of disease conditions. In some cases, ECs are also available from patients, and due to their authentic genetic background, these cells can be invaluable for research. However, obtaining patient-derived cells, especially for rare diseases, is challenging. In addition, ECs typically exhibit limited proliferation capabilities and have a restricted lifespan in culture, limiting their usability for extended studies. Moreover, in diseases caused by somatic mutations, the lesions from which these cells are sourced can contain a mix of both wild-type and mutant cells, adding complexity to the analysis process.

The use of primary ECs for disease modelling with genetic origins also poses some major challenges. First, the endogenous gene editing is challenging due to low efficiency of homology-directed repair, coupled with limited cell expansion and heterogeneity that limits clonal expansion [1]. Thus, in primary ECs, most in vitro work relies on siRNA transfection or viral transduction [2], the latter leading to unavoidable overexpression of the gene of interest under viral promoters resulting in expression of target proteins beyond physiological levels. siRNA transfection also comes with some limitations compared to endogenous CRISPR editing, including lower targeting specificity, transient effects, and expression leakage. Secondly, human umbilical vein endothelial cells (HUVECs) or other primary ECs obtained from patients are fully differentiated with tissue-specific properties and it is hard to critically observe the development of pathological features across the whole vasculature [3]. A potential solution to overcome the current limitations in primary EC models to mimic a genetic disease, is the development of induced pluripotent stem cell (iPSC) [4, 5] models. iPSCs are expandable, can be differentiated to the desired cell type, are easier to genetically modify and can have low batch to batch differences, allowing higher reproducibility, which is particularly important for drug screening [6].

Here we have developed and characterized human iPSC derived ECs (iECs) and used these generated cells as a proof-of-concept model to study TIE2-related venous malformations (VMs). TIE2 (gene name TEK), is a member of the receptor tyrosine kinase subfamily and is almost exclusively expressed in vascular ECs [7, 8]. TIE2 has a critical role in regulating blood vessel development and stability [9–12]. Its ligands are angiopoietin-1 (ANG1), angiopoietin-2 (ANG2) [13–15] and less investigated angiopoietin-4 [16–18]. ANG1 is a strong agonistic ligand inducing receptor multimerization, phosphorylation of specific tyrosine residues within the receptor's intracellular kinase domain, and TIE2 translocation to the specific subcellular domains [19–22]. In contrast, ANG2 is a competitive antagonist for TIE2 activating ligand ANG1 in blood ECs, except in some settings where it can act as a weak agonist [23, 24].

Physiological activation of TIE2 by its ligand initiates the PIK3CA/AKT/mTOR/FOXO1 signaling pathway [15, 17, 19, 20, 25–27] and this pathway plays a pivotal role in regulating EC survival, proliferation, migration, and vascular stability [9, 28]. On the other hand, the dysregulation of TIE2 has been implicated in various diseases, including cancer, cardiovascular disease, inflammatory disorders, acute kidney injury and VMs [29]. The latter are often caused by gain-of-function mutations in TIE2, TIE2^L914F^ substitution being the most common mutation.

Previous in vitro studies using viral overexpression in HUVECs have shown that TIE2^L914F^ causes a ligand-independent hyperphosphorylation, which leads to a persistent activation of TIE2 pathway independent of ANG1 [17, 30, 31]. In overexpressing HUVECs, TIE2^L914F^ shows abnormal cellular localization and response to ligand [30], an increased migration with loss of front-rear polarity [32] and dysregulation of genes involved in vascular development, cell migration and extracellular matrix (ECM) processing [31].

In this study, we implemented a CRISPR-Cas9 technique to generate a human iPSC line with an endogenous TIE2^L914F^ mutation. This approach enabled us to study the effects of the TIE2^L914F^ mutation on an endogenous level for the first time and in a model that doesn’t have a restricted lifespan in culture. Using iPSCs allowed us to study the differential effects of the L914F mutation in iPSCs differentiated to blood vessel iECs. We characterized the generated iECs and closely examined the impact of TIE2^L914F^ mutation on downstream TIE2 signaling and on the cellular transcriptomes. Employing a variety of in vitro and in vivo assays, we systematically investigated the effects of this mutation on iECs. When compared to the published histological data from clinical VM biopsies and previous disease models, iPSC TIE2^L914F^ recapitulated many characteristics of VMs, but also revealed alterations that are not observed in traditional models for VMs utilizing virally transduced primary ECs.

## Results

### Highly efficient generation of a TIE2^L914F^ iPSC line using the Xential CRISPR platform

**Fig. 1:**
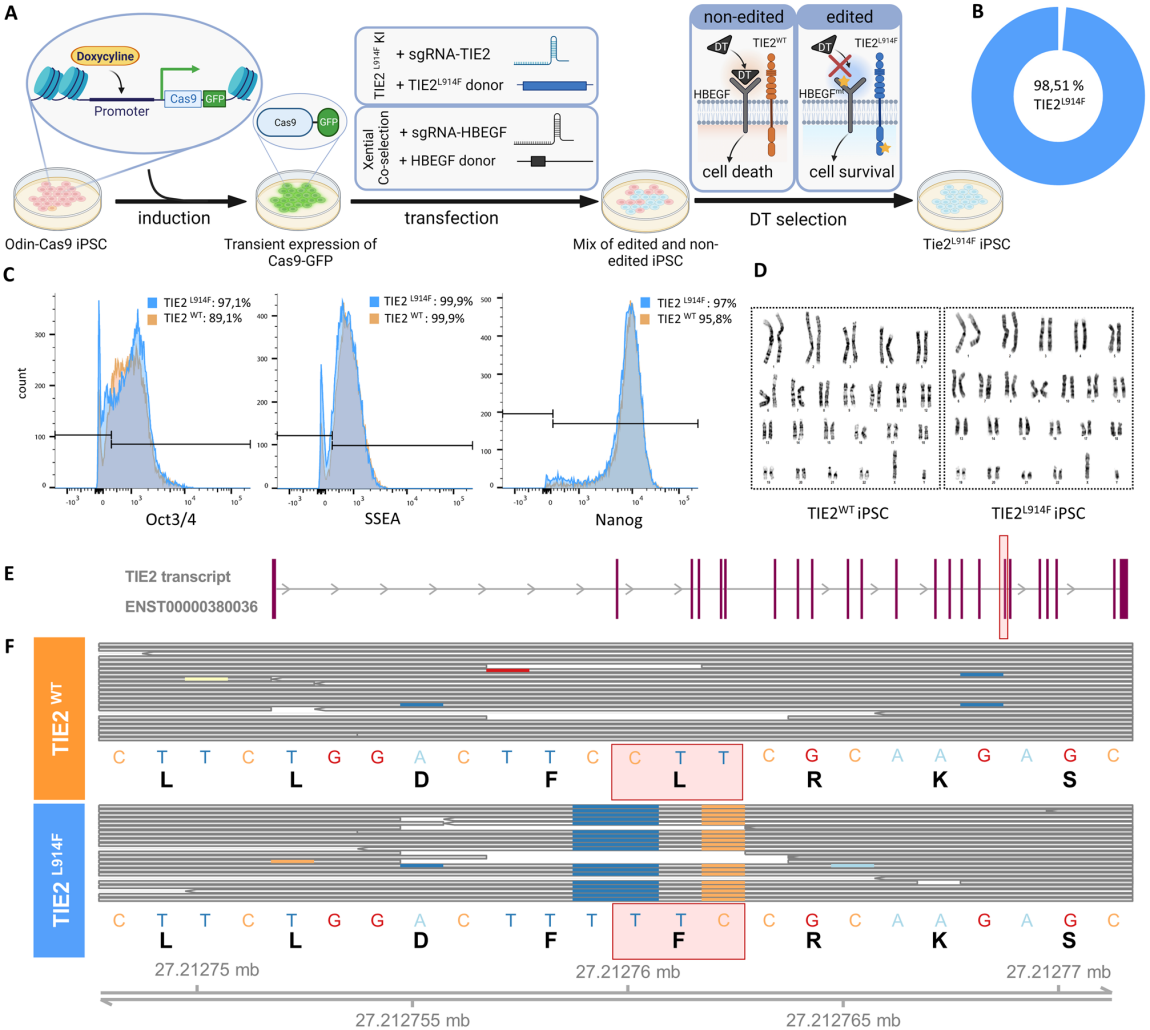
Highly efficient generation of a TIE2^L914F^ iPSC line using Xential CRISPR platform. **A** Step-by-step presentation of cell line generation. Step 1: induction of Cas9-GFP in OdinCas9-GFP iPSC; Step 2: transfection with target sgRNA and donor template for TIE2^L914F^ mutation and HBEGF co-transfection; Step 3: diphtheria toxin (DT) selection. **B** Amplicon-seq results showing the percentage of cells with TIE2^L914F^ mutation in the generated iPSC pool.** C** FACS results showing the expression of iPSC markers Oct3/4, SSEA and Nanog in TIE2^WT^ (orange) and TIE2^L914F^ (blue) iPSC line. **D** Karyotyping results of TIE2^WT^ and TIE2^L914F^ iPSC showing a healthy male phenotype.** E** Most transcribed TIE2 transcript in iECs differentiated from TIE2^L914F^ iPSC, as shown by RNA-sequencing. The edited exon is labelled with red square. **F** RNA transcripts of TIE2^WT^ and TIE2^L914F^ iEC lines, with the red square indicating the part where the mutation was introduced successfully. First row of each cell line shows the DNA codon and the second line for the amino acid translation

A TIE2^L914F^ iPSC line was successfully generated from a human iPSC line with an integrated doxycycline inducible Cas9-GFP system (ODInCas9 GFP iPSC), which has been shown to improve CRISPR editing [33]. The L914F mutation was strategically designed on a donor template and precisely incorporated into the TIE2 locus by sgRNA guided precise modification. To enhance the editing efficiency at the TIE2 locus, we applied the Xential co-selection strategy and co-introduced a diphtheria toxin (DT) resistance mutation at the endogenous HBEGF locus, which was then used for enriching edited cells with DT (Fig. 1A) [34]. We have previously shown that the mutation in HBEGF does not affect the differentiation abilities of iPSC [34]. This selection strategy allowed for the enrichment of edited cells and facilitated the generation of a highly homologous cell pool, predominantly comprised of successfully edited cells. Amplicon-seq was employed to assess the percentage of edited cells within the generated pool (Fig. 1B). The results demonstrated a remarkable success rate of over 98% in introducing the L914F mutation into the TIE2 locus. Due to a high editing efficiency and to minimize the likelihood of confounding effects associated with single-cell cloning, we utilized the pool of edited cells for subsequent experiments to enhance the reliability and reproducibility of our experimental platform [35].

We performed flow cytometry to evaluate the quality of generated iPSC lines. Results showed that both TIE2^L914F^ iPSC and TIE2^WT^ lines expressed similar levels of key pluripotency markers (SSEA-4, Nanog, Oct3/4) (Fig. 1C). To assess the genetic stability and chromosomal integrity of both TIE2^WT^ and TIE2^L914F^ iPSC lines, karyotyping analysis was performed. The karyotype analysis revealed that both cell lines exhibited a healthy male phenotype, devoid of any detectable chromosomal abnormalities or structural variations (Fig. 1D).

Next, we conducted a global RNA-Seq transcriptomic analysis on generated iPSC after their differentiation to vascular endothelial cells (iECs) to evaluate whether they have an edited TIE2 mRNA transcript. Results demonstrated that TIE2^L914F^ iECs have a full TIE2 transcript (Supp. Fig. 1) that carries the L914F mutation after differentiation, affirming the purity of the population (Fig. 1E, F).

Collectively, the comprehensive analyses presented herein underscore the successful generation and robust characterization of the TIE2^L914F^ iPSC line.

### iECs differentiated from TIE2^L914F^ iPSCs demonstrate a constitutively activated TIE2 pathway

**Fig. 2:**
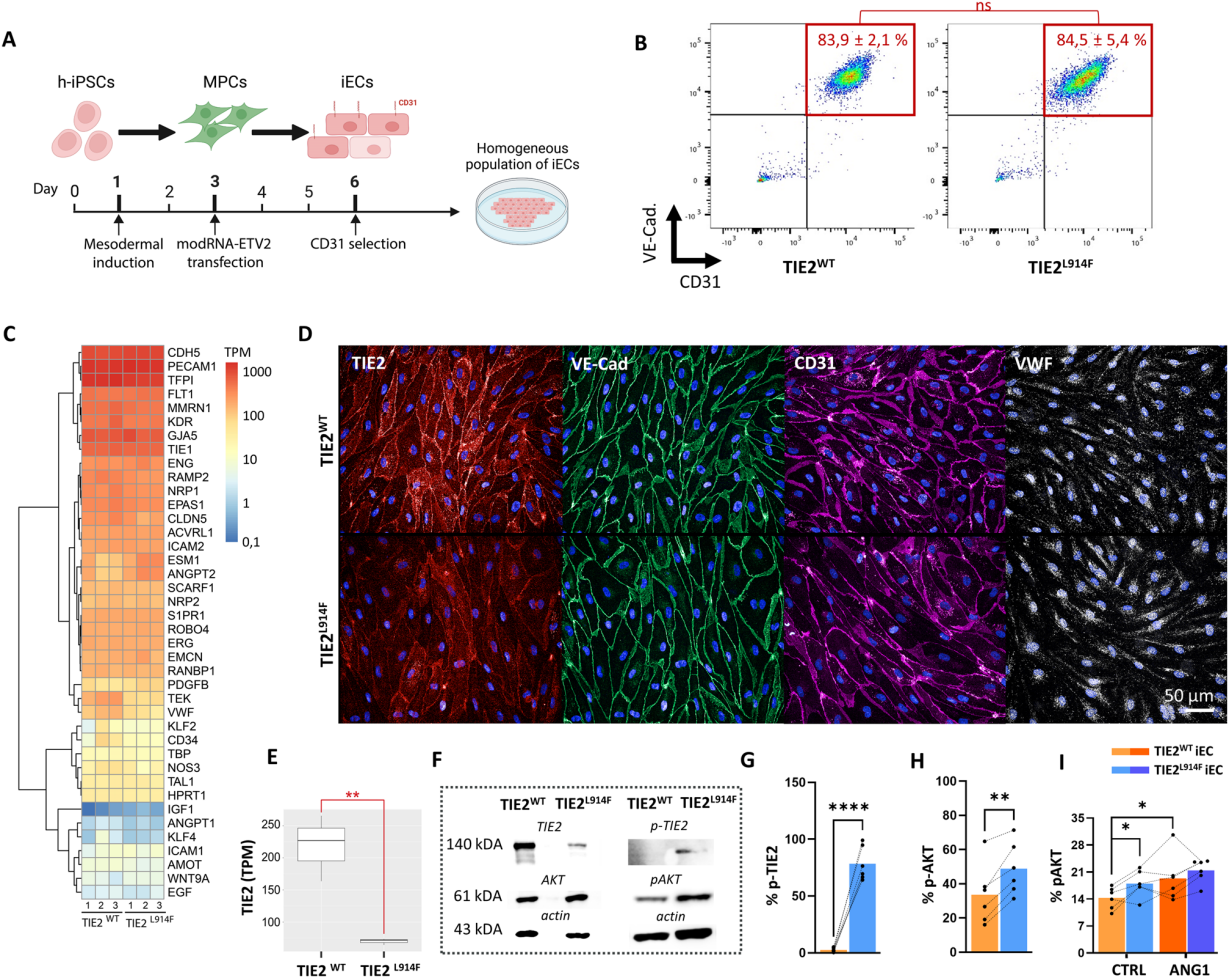
iECs differentiated from TIE2^L914F^ iPSCs demonstrate a constitutively activated TIE2 pathway. **A** Step-by-step presentation of a differentiation from iPSC to iECs through intermediate mesodermal progenitor cells (MPCs). **B** Flow cytometry of CD31 and VE-Cad in TIE2^WT^ and TIE2^L914F^ cell lines on day 6 of differentiation to iECs. n = 3. **C** Heat map and hierarchical clustering analysis of selected EC-specific genes. Colours represent transcripts per million (TPM). n = 3. **D** Immunofluorescence of TIE2 (red), VE-Cad (green), CD31 (purple) and VWF (white) in TIE2^WT^ (top) and TIE2^L914F^ (bottom) iECs. **E** Boxplot showing the TPM of TIE2 in generated iECs. **F** Western blot analysis of TIE2, pTIE2, AKT, pAKT and actin in iECs. **G** Quantification of western blot signal in (**F**) showing the percentage of phosphorylated TIE2 in iECs. n = 6. TIE2^WT^ (orange) and TIE2.^L914F^ (blue) iECs. **H** Quantification of western blot signal in (**F**) showing the percentage of phosphorylated AKT in iECs. n = 6. **I** Quantification of AKT phosphorylation assay with and without the ANG1 induction. n = 6. n represents a number of separate iEC differentiations per group. Dots represent an average value of each biological replicate, separate iEC differentiations. Dots that belong the same biological replicate are linked. n = 3.*p < 0,05, **p < 0,01, ***p < 0,001

We differentiated iPSC cells into iECs to assess the effects of the L914F mutation on generated lines (Fig. 2A). This differentiation process involved mesoderm specification via Wnt activation, followed by endothelial differentiation with overexpression of the EC-specific transcription factor ETV2, through modified RNA (modRNA) transfection [36]. Differentiation efficiency was consistent between TIE2^WT^ and TIE2^L914F^ cell lines, with flow cytometry results demonstrating over 84% of CD31/VE-Cadherin (VE-Cad) double-positive cells before CD31 selection (Fig. 2B). Bulk RNA-Seq analysis revealed that both TIE2^WT^ and TIE2^L914F^ iECs expressed typical EC markers, confirming their successful differentiation into the EC lineage (Fig. 2C). Immunocytochemistry (ICC) further validated the endothelial phenotype, as evidenced by positive staining for EC markers, including TIE2, VE-Cad, CD31, and Von Willebrand Factor (VWF), in both TIE2^WT^ and TIE2^L914F^ iECs (Fig. 2D). The transcriptomic data indicated that the cells display a heterogenous endothelial specification with a stronger arterial-like identity (Supp. Fig. 3).

To evaluate the functional consequence of the L914F mutation in our iECs, we investigated the TIE2 pathway including the downstream phosphorylation of AKT as well as transcriptome changes. Western blot analysis revealed higher phosphorylation levels of both TIE2 and AKT in TIE2^L914F^ iECs (Fig. 2F–H), indicating a gain-of-function effect as expected. This finding was corroborated by an AKT phosphorylation assay (Fig. 2I), which confirmed increased AKT phosphorylation in iECs downstream of TIE2^L914F^ when compared to TIE2^WT^ iECs. Upon stimulation of the TIE2 pathway with ANG1, the difference in phosphorylation levels between TIE2^WT^ and TIE2^L914F^ iECs decreased, that indicated saturated activation of TIE2^L914F^. These results corroborate the previous reports in which TIE2^L914F^ overexpression led to the ligand-independent constitutive TIE2 signaling pathway activation [30].

Interestingly, we observed decreased endogenous TIE2 expression levels in the TIE2^L914F^ iEC line, supported by RNA-Seq data of TIE2 mRNA expression (Fig. 2E) and protein-level analyses (Western blot and immunocytochemistry) (Fig. 2F). Previous studies on the TIE2^L914F^ mutation primarily used HUVECs with overexpressed TIE2^L914F^ under constitutive viral promoters [30, 31, 37–39]. In contrast, using an iPSC approach we were able to uniquely explore the effects of TIE2^L914F^ at the endogenous expression levels using a knock-in mutation in TIE2 locus.

Previously we found that TIE2^L914F^ when overexpressed in HUVECs has an incomplete response to ANG1, is retained in the endoplasmic reticulum and is auto-phosphorylated in different subcellular compartments compared to TIE2^WT^ [40]. To see whether TIE2^L914F^ in iECs similarly affects the spatial distribution and activation of TIE2 upon ANG1 stimulation, TIE2^WT^ and TIE2^L914F^ iECs were left unstimulated or stimulated with ANG1, fixed and stained with total and P-TIE2 antibodies (Supplemental Fig. 5). While TIE2^WT^ showed an expected clustering on the membrane upon ANG1 stimulation, TIE2^L914F^ subcellular localization in iECs was almost unchanged. Levels of P-TIE2 were too low to detect in our setting by immunofluorescent staining, thus we were not able to investigate its localization.

### TIE2^L914F^ iECs results in gene expression profiles implicated in cell migration and angiogenesis

**Fig. 3:**
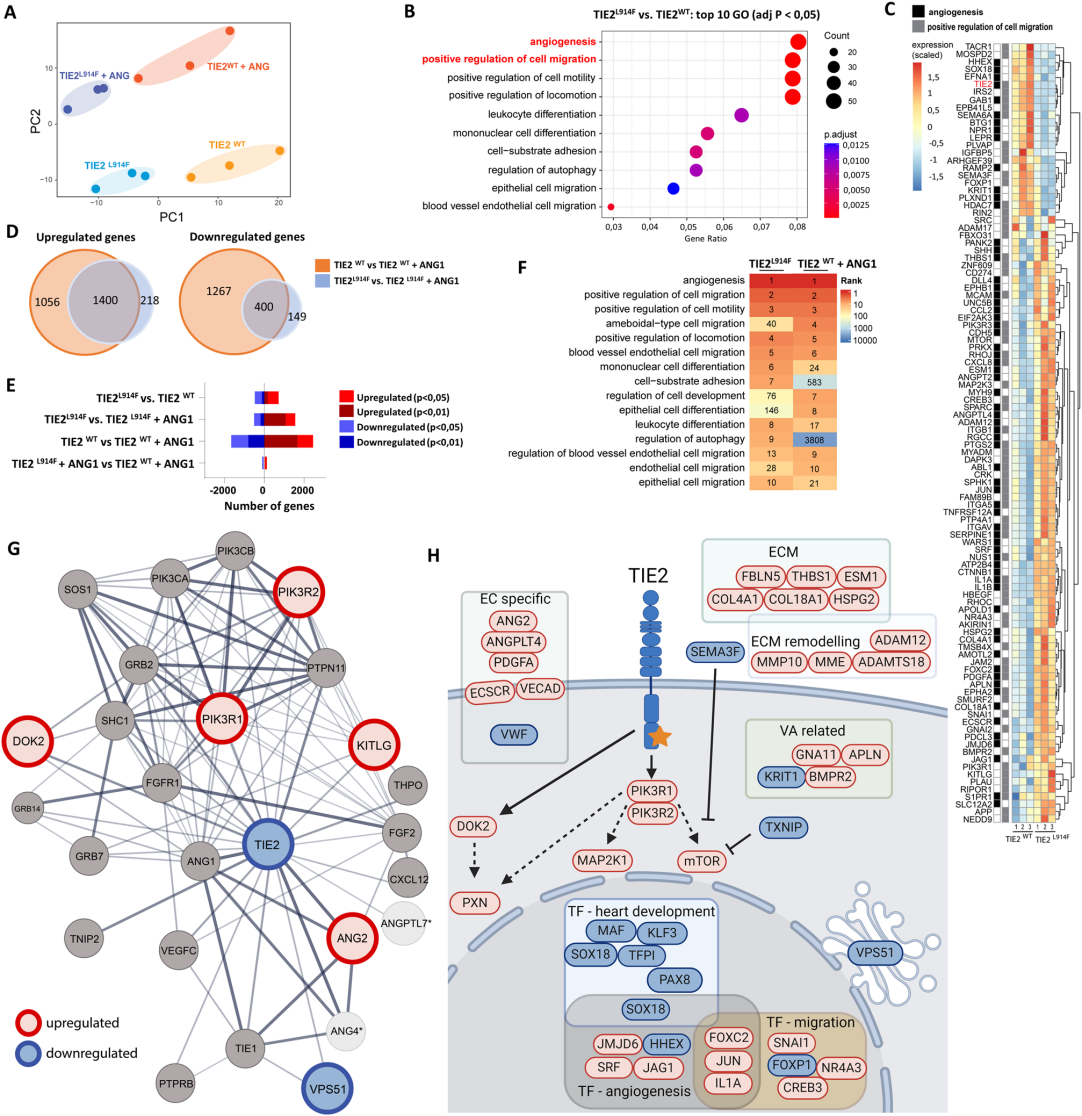
TIE2^L914F^ iECs results in gene expression profiles implicated in cell migration and angiogenesis. **A** Principal component analysis (PCA) of samples from TIE2^WT^ and TIE2^L914F^ iECs, ± ANG1. **B** Top ten upregulated gene ontologies in TIE2^L914F^ vs. TIE2^WT^ iECs comparison. Colours represents the significance and size represents the number of significant genes for each gene ontology. **C** Heat map of gene expression (scaled TPM-values) and hierarchical clustering analysis of genes included in gene ontologies for angiogenesis and positive regulation of cell migration for TIE2^WT^ and TIE2^L914F^ iECs. **D** Cross section of genes that were up-/down regulated upon ANG1 induction in both cell lines and have adjP < 0,05; TIE2^WT^ (orange) and TIE2^L914F^ iECs (blue). Numbers of up-/down regulated genes are written in each cross section. **E** Graphical presentation of a number of up-/down regulated differentially expressed genes, from each iEC comparison.** F** A comparison of top ten upregulated gene ontologies in TIE2^L914F^ and TIE2^WT^ + ANG1 iECs, when analysed against TIE2^WT^ iECs. The rank numbers represent the order of gene ontologies, with 1 indicating the highest significance. **G** Known TIE2 protein interaction partners (STRING) and changes in their mRNA expression based on TIE2^L914F^ vs. TIE2^WT^ comparison. Genes with p < 0,05 were coloured with red (upregulated) or blue (downregulated). Genes without significant difference are coloured grey. *No data. **H** Selected differentially expressed genes from TIE2^L914F^ vs. TIE2^WT^ iEC comparison that are important for regulation of angiogenesis, migration or heart development, are related to VAs, are typically expressed in EC or are a part of TIE2 downstream pathway. n = 3 iEC differentiations per group. Genes with p < 0,05 were coloured with red (upregulated) or blue (downregulated)

To gain deeper insights into the molecular distinctions and potential effects of the TIE2^L914F^ mutation in iEC models, we conducted bulk RNA-Seq analysis on both TIE2^L914F^ and TIE2^WT^ iEC lines, with and without the induction of the TIE2 ligand ANG1. This allowed us to compare the effects of physiological TIE2 activation and ligand independent activation caused by L914F mutation and enabled us to study whether addition of ligand results in quantitatively or qualitatively different responses in the TIE2^L914F^ iEC line. To ensure robustness and accuracy, we employed biological replicates from three separate differentiations for each condition, resulting in four distinct sample groups: TIE2^L914F^, TIE2^WT^, TIE2^L914F^ + ANG1, and TIE2^WT^ + ANG1. The Principal Component Analysis (PCA) plots demonstrated that biological replicates for each of the four samples exhibited close grouping, while different conditions separated from each other (Fig. 3A).

To understand the effects of L914F on the cell transcriptome, we first conducted a gene ontology analysis for TIE2^L914F^ vs. TIE2^WT^ iECs (Fig. 3B). The results revealed that the two most upregulated gene ontologies were associated with angiogenesis and positive regulation of cell migration. Furthermore, multiple upregulated gene ontologies were linked to cell migration. In Fig. 3C, the heatmap displays all differentially expressed genes involved in angiogenesis and the positive regulation of cell migration.

Next, we investigated the effect of ANG1 stimulation within each cell line; TIE2^L914F^ vs. TIE2^L914F^ + ANG1 and TIE2^WT^ vs. TIE2^WT^ + ANG1. Our findings revealed that upon ANG1 stimulation, the up- and down- regulated genes in TIE2^L914F^ iECs largely coincided with those upregulated in TIE2^WT^ iECs (Fig. 3D). However, the number of differentially expressed genes upon ANG1 stimulation was higher in the TIE2^WT^ iEC line (Fig. 3E). Consequently, the addition of ANG1 had a less pronounced effect on the mutant cells. To evaluate whether that is due to a constitutively activated TIE2 pathway in the TIE2^L914F^ iECs, we assessed whether gene ontologies upregulated in TIE2^L914F^ iECs in the absence of ANG1, resemble those in TIE2^WT^ induced with ANG1. By comparing the rankings of the top 10 upregulated gene ontologies in these samples, we found that most upregulated gene ontologies are overlapping (Fig. 3F). The most notable differences were observed in the GO terms of upregulation of cell-substrate adhesion and regulation of autophagy, which were specifically upregulated only in TIE2^L914F^ iECs. Interestingly, while both TIE2^L914F^ and TIE2^WT^ lines had multiple upregulated pathways upon ANG1 induction only TIE2^L914F^ iEC line had significantly downregulated gene ontologies following ANG1 induction (Supp. Fig. 2).

To investigate whether any of the genes that code for proteins known to interact with the TIE2 receptor exhibited differential expression in TIE2^L914F^ iECs (Fig. 3G), we extracted the network of known TIE2 protein interactors from the STRING database [41]. The results indicated that despite lower TIE2 expression in TIE2^L914F^ iECs, genes downstream of the TIE2 pathway appeared to be transcriptionally upregulated, as evidenced by the differential upregulation of PIK3R1 and PIK3R2. To gain further insight, we examined whether there were more differentially expressed genes downstream of PIK3CA, revealing that mTOR and MAP2K1 were also upregulated (Fig. 3H). Additionally, ANG2, a known TIE2 inhibitor, displayed a small but significant upregulation. The average expression of ANG2 was 1,2 times higher than in TIE2^WT^ iECs. Interestingly, previous research on HUVECs TIE2^L914F^ overexpressed models showed a decrease in ANG2 expression [31]. To investigate this further we measured protein levels of ANG2 using ELISA and the results showed a slightly (statistically non-significant) higher expression levels of ANG2 in TIE2^L914F^ iECs. We subsequently investigated the localization of FOXO1, a known regulator of ANG2 expression [42, 43] that is located to the cytosol upon phosphorylation induced by TIE2/AKT signaling [44]. Our observations revealed a significant reduction in FOXO1 localization within the nucleus in TIE2^L914F^ iECs, also indicating an active TIE2 pathway (Supp. Fig. 4a). When compared to HUVEC overexpression models, nuclear levels of FOXO1 were found to be higher in TIE2^L914F^ iECs (Supp. Figure 4b).

Since we observed lower mRNA TIE2 levels in TIE2^L914F^ iECs we further investigated whether any transcription factors (TF), known to regulate TIE2 expression, are dysregulated. Results revealed differences in multiple TFs that are known or predicted to bind TIE2 promoter or enhancer regions according to GeneCards [45, 46], such as MAFF, POLR2A, KLF7, BHLHE40, AHR, POLR2A, MAF, ZNF366, ZNF561, BCL6B. There were also multiple upregulated TFs in TIE2^L914F^ iECs (Fig. 3H) that are linked to cell migration and angiogenesis, while several downregulated TFs were associated with heart development, which may be relevant to ANG1 gene deficient mice where cardiac problems were observed [47, 48].

Previous research using retrovirally transduced HUVECs indicated a dysregulation of genes encoding proteins related to ECM and its remodeling, such as MMPs and ADAMs [2, 31, 40]. When we investigated expression of ECM related genes in our samples, we noticed multiple changes (Fig. 3H). There was a significant dysregulation of COL4A1 COL18A1, THBS1, FBLN5 and HSPG2. Similarly, there was a significant upregulation of proteins important for ECM remodeling such as MMP10, ADAM12 and ADAMTS18.

To get insights if VMs and other types of vascular anomalies (VAs) are mechanistically linked to each other we next investigated genes that are known to cause or be altered in VAs. Indeed, we observed dysregulation of KRIT1, related to cerebral cavernous malformations (CCM) [49], APLN, related to lymphatic malformations [50], GNA11 [51] related to capillary malformations and BMPR2, related to arteriovenous malformations and hereditary pulmonary arterial hypertension (HHT) [52].

### TIE2^L914F^ iECs demonstrate enhanced migratory capacity and normal proliferation rates in vitro

**Fig. 4:**
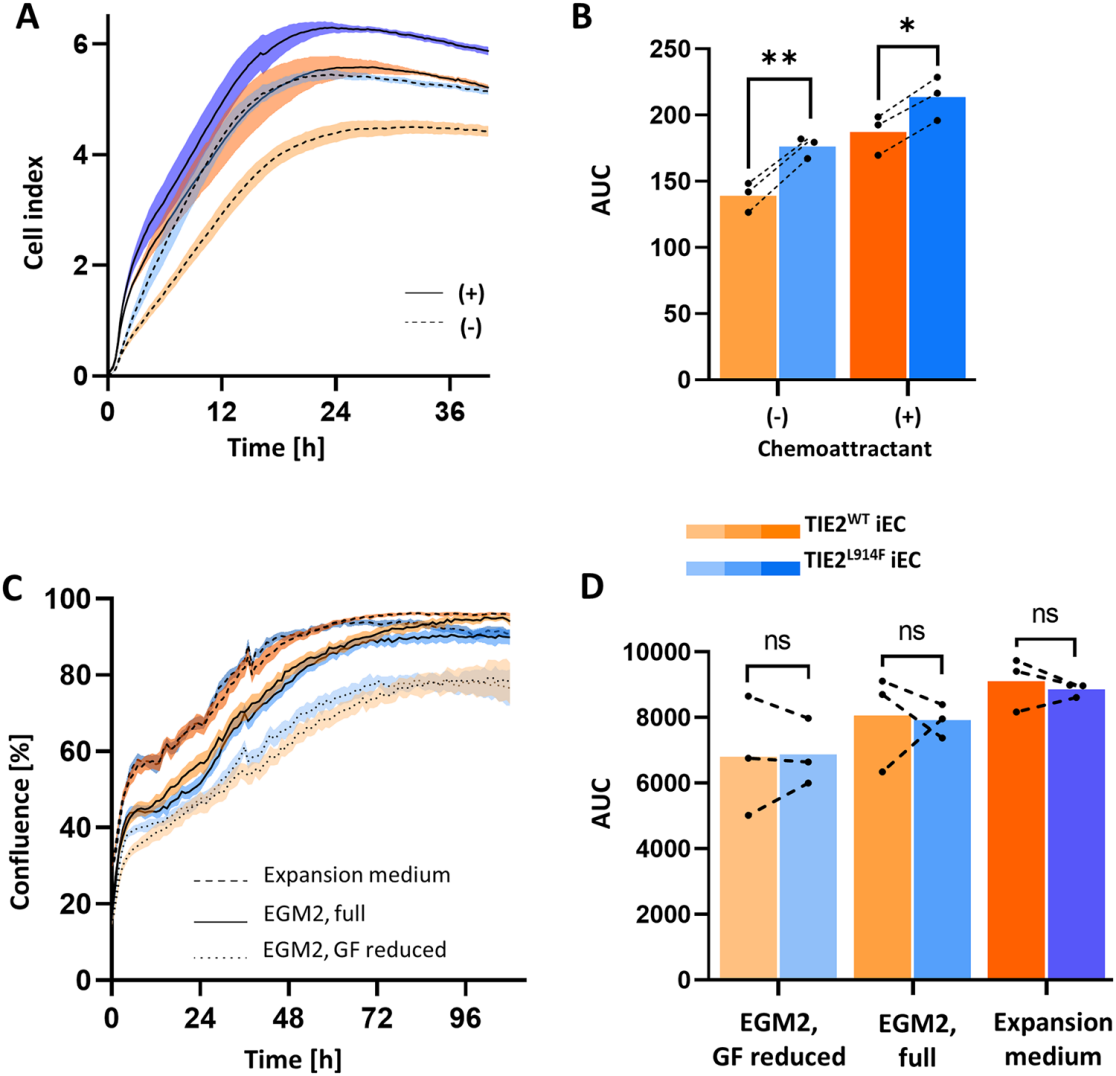
Tie2^L914F^ iECs demonstrate enhanced migratory capacity and normal proliferation rate in vitro*.* **A** Curves showing the migration of cells in time with ( +) and without (–) the presence of chemoattractant (10% serum). **B** Quantification of area under the curve (AUC) for migrated cells shown in (**A**). n = 3. **C** Curves showing the proliferation rate of TIE2^WT^ and TIE2.^L914F^ iECs in time, by measuring cell area with Incucyte. Graph shows curves of cells in expansion medium, fully supplemented EGM2 medium and starvation medium (EGM2 with reduced growth factors, GF). n = 3. **D** Quantification of area under the curve (AUC) from proliferation data in (**C**). n = 3. n represents a number of separate iEC differentiations per group. Error bars represent mean ± SE. Dots represent an average value of each biological replicate, separate iEC differentiations. Dots that belong to the same biological replicate are linked. n = 3.*p < 0,05, **p < 0,01, ***p < 0,001

To gain further insights into the characteristics of the TIE2^L914F^ iEC line, we conducted an in vitro migration assay using the real-time cell analysis instrument (Fig. 4A, B). This assay allowed us to compare the migration abilities of generated iEC lines in the presence and absence of a chemoattractant, which in this case was 10% EGM2 serum. The results of the migration assay showed that the TIE2^L914F^ iECs demonstrated significantly higher migratory capabilities compared to the TIE2^WT^ iECs, regardless of the presence or absence of the chemoattractant. Notably, the migration values obtained for the TIE2^L914F^ iECs in the absence of chemoattractant were similar to the values obtained for the TIE2^WT^ iECs in the presence of the chemoattractant. These findings suggest that the L914F mutation confers a hypermigratory phenotype to the ECs, leading to enhanced cell migration capacity even in the absence of external chemoattractant stimuli. Additionally, the similarity in migration values between TIE2^L914F^ iECs without chemoattractant and TIE2^WT^ iECs with chemoattractant implies that the L914F mutation may cause constitutive activation of migratory pathways in ECs, bypassing the requirement for external cues. These findings corroborate the transcriptomics data, where we observed upregulated migration related pathways in TIE2^L914F^ iEC line.

To investigate the impact of the L914F mutation on EC proliferation, we conducted a proliferation assay using imaging-based technology. The proliferation of cells was monitored over time under different medium conditions to ensure that any observed differences were not influenced by variations in media composition. We tested starvation medium (0,5% EGM2), normal medium (2% EGM2), and expansion medium optimized for ECs proliferation. The results obtained (Fig. 4C, D) indicated no significant difference in cell proliferation between the TIE2^L914F^ and TIE2^WT^ iEC lines across all tested medium conditions. This suggests that the L914F mutation does not exert a significant effect on the proliferation rates of iECs.

### TIE2^L914F^ iECs showed larger cell area than TIE2^WT^ iECs and were less sensitive to high shear stress in a microfluidic model

**Fig. 5:**
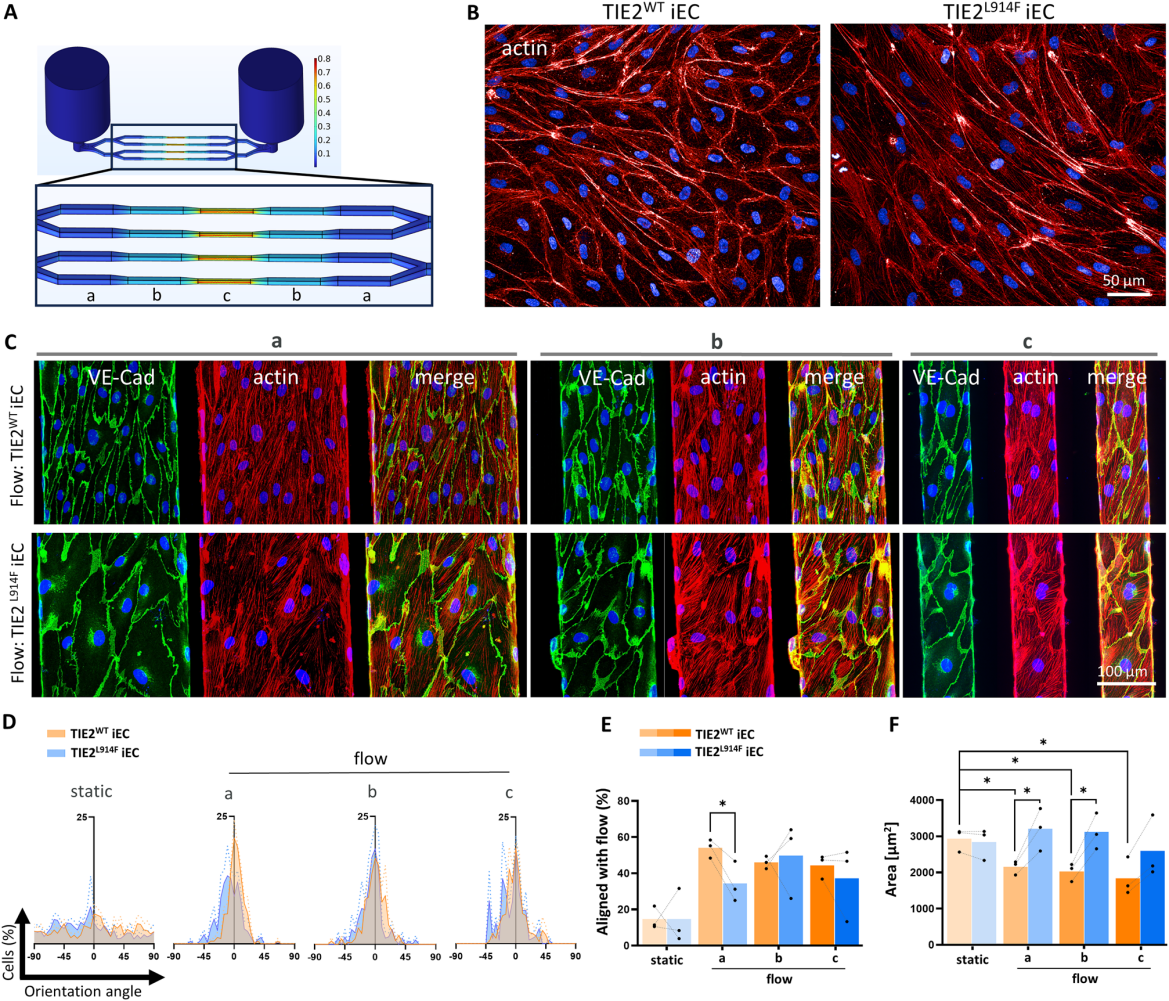
TIE2^L914F^ iECs showed larger cell area than TIE2^WT^ iECs and were less sensitive to high sheer stress in a microfluidic model. **A** Schematic presentation of a microchannel unit and different cross-sections of microfluidic vessels shown in a, b and c. Section “a” having the lowest sheer stress and “c” the highest. **B** Immunofluorescence of actin cytoskeleton in TIE2^WT^ (left) and TIE2^L914F^ (right) iECs without the presence of flow, static.** C** Immunofluorescence of TIE2^WT^ (top) and TIE2^L914F^ (bottom) iECs under flow shear stress conditions a, b and c; VE-Cad (green), actin/phalloidin (red). **D** Curves showing the distribution of cell orientation in the direction of flow, represented by 0^o^, in static and different flow conditions (a,b,c) in TIE2^WT^ (orange) and TIE2.^L914F^ (blue) iECs. **E** Quantification of cells oriented in flow (0° ± 5°). n = 3. **F** Quantification of average cell area under different static/flow conditions. Dots represent an average value of each biological replicate, separate iEC differentiations. Dots that belong to the same biological replicate are linked. n = 3.*p < 0,05, **p < 0,01, ***p < 0,001

VMs belong to a group of slow-flow vascular malformations, thus we tested the effect of flow on cellular morphology and orientation of TIE2^L914F^ and TIE2^WT^ iECs. To investigate the influence of flow shear stress in blood vessel-mimicking condition, we employed microfluidic platform. Providing a graduated shear stress gradient denoted as levels (a)—0,1 Pa, (b)—0,2 Pa, and (c)—0,65 Pa (Fig. 5A). In the absence of flow, TIE2^L914F^ iECs exhibited a deviation in actin organization (Fig. 5B), but no significant difference in cell length or area.

To quantitatively assess cell orientation in response to flow, we measured the longest diameter of each imaged cell and compared their orientations to the flow direction, defined as 0°, under each shear stress condition (a, b, c) (Fig. 5D). A significantly lower number of TIE2^L914F^ iECs aligned with the flow direction compared to TIE2^WT^ iECs under the lowest shear stress condition (a), which may mimic the low flow condition in patient lesions. A similar trend was observed under higher shear stress conditions, although the difference did not reach statistical significance.

Furthermore, our cell analysis revealed a significant decrease in area of TIE2^WT^ iECs when in flow but not in TIE2^L914F^ iECs, with TIE2^L914F^ iECs showing a larger cell area than TIE2^WT^ in all three shear stress conditions.

### TIE2^L914F^ iECs form dilated blood vessels in vivo in SCID mouse transplantation model

**Fig. 6:**
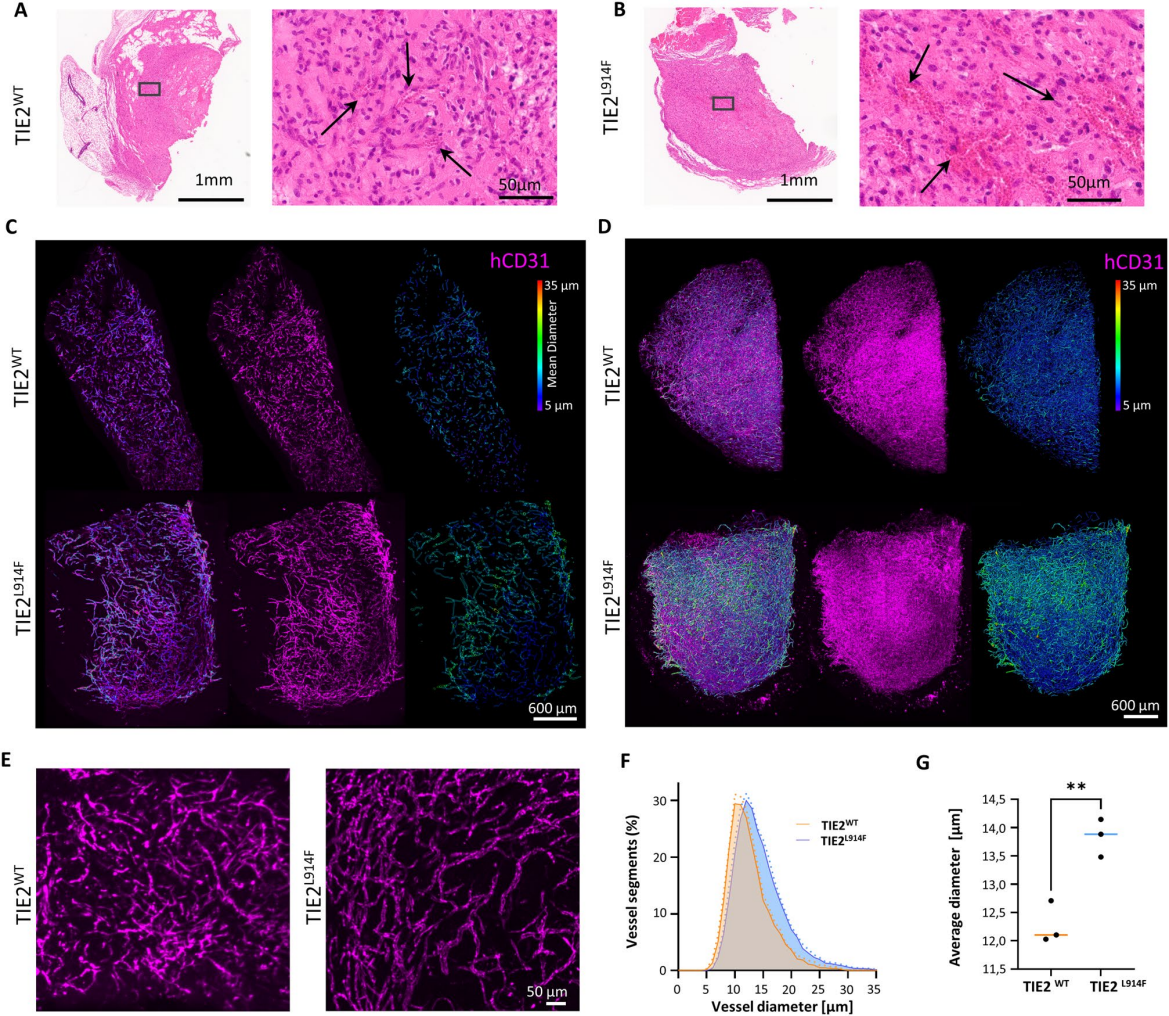
TIE2^L914F^ iECs form dilated blood vessels in vivo in SCID mouse transplantation model. H&E stained sections of **A** TIE2^WT^ iECs and **B** TIE2^L914F^ iECs xenograft explants. The left panel displays an overview of the entire section, while the right panel provides a magnified view of the central area of the explant. Arrows are indicating areas with RBCs. **C** z-stacks of xenograft explants captured with light microscopy, depicting the hCD31 signal (purple, middle) and vessel segments (right) obtained through filament tracer analysis from hCD31 signal. The colour scheme on the right indicates the mean diameter of the vessel segments. Images on the left display a merge view of these signals. **D** 3D images of xenograft explants showing the hCD31 (purple, middle) signal and 3D architecture of generated vessels (right). Vessels were visualised from vessel segments data shown in (**C**) and the colour scheme indicates the mean diameter of the vessels. Images on the left display a merge view of these signals. **E** Magnified view of hCD31 signal showing the formation of vessels in xenograft explants. Vessels in TIE2^WT^ iEC samples (right) appear wider. **F** Data obtained with filament tracer analysis showing the distribution of vessel diameters in xenograft explants from TIE2^WT^ iECs and TIE2^L914F^ iECs (n = 3). **G** Quantification of average vessel diameter in xenograft explants (n = 3). Dots represent an average diameter value for each xenograft explant. *p < 0,05, **p < 0,01, ***p < 0,001

In prior investigations, the HUVEC TIE2^L914F^ model demonstrated an in vivo phenotype of forming VM resembling lesions when transplanted in severe combined immunodeficient (SCID) mice [37, 40, 53]. To investigate the competency of iECs model in forming similar vascular lesions, TIE2^WT^ or TIE2^L914F^ iECs were mixed with mesenchymal stem cells (MSCs) in a 1:1 ratio and implanted into SCID mice for a duration of 7 days. Grafts were investigated using histological methods (Fig. 6A, B). In addition, iECs formed vascular structures within the grafts were stained using human-specific CD31 (Fig. 6C, D). Histological analysis revealed that TIE2^L914F^ explants exhibited a higher number of red blood cells (RBC), suggesting of enhanced perfusion within those explants (Fig. 6A, B). To gain insight into the three-dimensional architecture of the TIE2^WT^ and TIE2^L914F^ iECs formed structures, xenograft explants were imaged with light-sheet microscopy. Images were analysed with filament tracer module that recognized CD31 signal in each z-stack as vessel segments (Fig. 6C), followed by reconstruction of 3D structures generated by iECs (Fig. 6D). Analysis of generated data indicated that both cell lines successfully formed vascular structures, although TIE2^L914F^ vessels displayed a larger vessel diameter compared to TIE2^WT^ (Fig. 6E–G).

## Discussion

iECs have emerged as a promising model for studying vascular biology and disease, offering several advantages over traditional primary cell lines such as HUVECs. This transition reflects a growing recognition of the unique benefits iECs bring to the field, enhancing our understanding of ECs’ physiological functions. As iPSCs are amenable to genetic manipulation, including the introduction of disease-associated mutations or gene editing techniques like CRISPR-Cas9 [54], the iECs also provides efficient platform to investigate diseases with genetic origins [55]

In this study, we established an iPSC-derived model to investigate the TIE2^L914F^ mutation commonly found in VMs. The TIE2^L914F^ iPSC line was successfully generated and characterized using Xential [34], a CRISPR-mediated genome editing coupled with DT selection, that helped us achieve a high editing efficiency. Notably, our approach involved working with an enriched pool of edited cells rather than single-cell clones, a choice that offers several advantages. Single-cell clones are prone to genetic and phenotypic variability due to clonal selection and genetic drift, resulting in clonal artefacts [56]. In contrast, a cell pool comprises cells with diverse characteristics, mitigating individual variations. This strategy more closely mimics the natural in vivo variability of cell types and phenotypes, capturing the complexity of cellular interactions and responses and enhancing experimental efficiency, especially in high-throughput applications [35].

To characterise the generated cells, we differentiated them to vascular iECs and assessed whether our iPSC derived TIE2^L914F^ iECs have an upregulated TIE2 pathway, as previously described in the literature. [2, 17, 30, 32]. We performed an AKT phosphorylation assay, western blot analysis of pTIE2 and pAKT and RNA-seq. Our results indeed showed an increased level of AKT and TIE2 phosphorylation at the protein level and an upregulation of gene transcription downstream of TIE2 pathway, such as PIK3R1, PIK3R2, mTOR and MAP2K1. This indicated that our iPSC derived TIE2^L914F^ iECs have a dysfunctional TIE2 and that the mutation is indeed causing the activation of TIE2 pathway in the absence of ANG1.

Western blot and RNA-seq results also revealed an unexpectedly low level of TIE2 protein and mRNA transcript in the TIE2^L914F^ iECs. This event has not been observed before as all previous in vitro research on TIE2^L914F^ mutation was done on HUVECs that overexpressed TIE2^L914F^ receptor under a viral promoter. A possible explanation for this could be that a negative feedback loop caused by the constitutive activation of the TIE2 pathway occurred to downregulate TIE2^L914F^ mRNA expression. RNA-seq indeed showed a dysregulation of multiple TFs linked to TIE2 expression, however, further evidence would be needed to confirm this hypothesis. Downregulation of TIE2 gene expression is also an interesting observation from a point of view of VM pathogenesis as decreases in TIE2 may contribute to vascular dysmorphogenesis [9, 11, 57].

In addition, RNA-seq results showed a differentially upregulated expression of ANG2, a context-dependent agonistic ligand that can interfere with TIE2 activation by ANG1 in blood ECs. Interestingly, previous research on HUVEC TIE2^L914F^ overexpressed model showed the opposite effect on ANG2 as they observed a decrease in its expression [31].

The regulation of ANG2 transcription by the FOXO1 transcription factor is well-established [42, 43], and it is known that the TIE2 pathway can induce the phosphorylation of FOXO1, leading to its translocation from the nucleus to the cytosol and subsequent inactivation [44]. Consequently, differences in ANG2 expression observed between experimental conditions may be attributed to the overexpression of TIE2^L914F^ in the HUVEC model resulting in complete clearance of FOXO1 from the nucleus, indicative of its inactive state. Conversely, in TIE2^L914F^ iECs, FOXO1 was only partially cleared from the nucleus, suggesting the presence of remaining active FOXO1 capable of influencing ANG2 transcription. These findings underscore the intricate regulatory mechanisms involving FOXO1 and TIE2 signalling in the modulation of ANG2 expression and angiogenic processes and could further indicate an existence of a negative feedback loop that downregulates TIE2 expression.

Gene ontology analysis highlighted dysregulated pathways linked to angiogenesis and cell migration in TIE2^L914F^ iECs. A recent study on patient lesions with TIE2^L914F^ mutation involved a transcriptomics data that corroborated our findings by demonstrating upregulation of angiogenesis/blood vessel development and positive regulation of cell migration [58]. Moreover, the study highlighted an upregulation of genes involved in ECM organization and remodelling, aligning with our identification of the related gene ontology term, "cell-substrate adhesion." These included ADAMTS18, MMP10, MME and various ECM proteins, which have been observed in patient samples before [58, 59]. This consistency underscores the relevance and utility of our model for investigating vascular pathologies linked to TIE2 mutations. Regrettably, the current transcriptomic data from patient VM lesions is not complete, and human VM EC-specific data is not available thus hindering comparison of the expression levels of TIE2 and ANG2, among other EC expressing genes, as depicted in Fig. 3G, H of our study.

In a previous study, which utilized transcriptomics data from a HUVEC TIE2^L914F^ model [31], similarly demonstrated upregulation of cell migration and ECM remodelling, while vascular development was predominantly downregulated. Upon comparing HUVEC TIE2^L914F^ and TIE2^L914F^ iEC data in more detail [31], we identified three genes that were significantly downregulated in both; VWF, CITED2 and DEPP, while ANG2, ANKRD1, THBS1 and MMP10 were upregulated in TIE2^L914F^ iECs but downregulated in TIE2^L914F^ HUVECs. Similarly, when comparing our data to the list of upregulated genes in the HUVEC model [31], three genes, namely ADAMTS18, APOLD1, and IL32, were found to be significantly upregulated in both studies, whereas TXNIP, SEMA3F, and IGFBP5 were downregulated in TIE2^L914F^ iECs. Additionally, a longer list of genes was significantly upregulated in human lesions with verified TIE2^L914F^ mutation [58] compared to TIE2^L914F^ iECs, including MYC, JUN, UAP1, TPPP3, CSRNP1, DUSP1, FTL, CTSB, PIK3R1, CDKN1A, MCL1 and FNDC3B. While PDK4, AHR, MAF and ARRDC3 were downregulated in iECs but upregulated in patient tissue. Nevertheless, further investigations are warranted to validate the significance of these findings and propose a molecular mechanism underlying VMs.

Next, we compared available transcriptomic data from TIE2^L914F^ iECs, HUVECs TIE2 ^L914F^ model and VM lesions to identify VM relevant genes common in all. Notably, only one gene, BHLHE40, was significantly upregulated in all three studies. It is crucial to acknowledge that a comparison could yield additional overlapping genes and differences if full transcriptomics data were available for all experiments.

To further study the characteristics of generated iECs in vitro we conducted migration and proliferation assay. Migration assay displayed increased migration abilities in TIE2^L914F^ iECs with and without the presence of chemoattractant, which aligns with RNA-seq data. Controlled cell migration is pivotal for correct tissue morphogenesis. The exact importance of cell motility in VM pathogenesis is currently unknown, however, continuously active, ECs migration may be involved in the growth of vascular lesions in VMs in the absence of EC proliferation [2, 31, 40]. This also aligns with results from our proliferation assay, that showed no difference in proliferation between the iEC lines.

We next followed the spatial distribution of TIE2 upon ANG1 induction. ANG1 ligand-binding induces TIE2 translocation and activation in specific subcellular domains resulting in cell compartment-dependent downstream signalling [17, 21]. In our previous study [40] using retrovirally transduced HUVECs we found that in contrast to TIE2^WT^, L914F mutation results in TIE2 protein retention in the endoplasmic reticulum, autophosphorylation in the Golgi, punctuate clustering and incomplete translation as a response to ANG1. Similarly, to the retrovirally transduced HUVECs, TIE2^L914F^ iECs showed incomplete clustering/translocation to the cell–cell contacts upon ANG1 induction. In contrast to TIE2^L914F^ HUVECs, perinuclear TIE2 accumulation was not a prominent feature of TIE2^L914F^ iECs. As a technical limitation, we were not able to investigate TIE2 phosphorylation state in specific subcellular compartments in iPSC-derived iECs due limited sensitivity and specificity of pTIE2 antibodies to detect activated TIE2 without overexpression.

As VMs belong to a group of slow flow vascular malformations, we implemented microfluidic plates to study the effect of flow shear stress on TIE2^L914F^ and TIE2^WT^ iECs. TIE2^L914F^ iECs demonstrated defective orientation in flow direction, particularly evident under low shear stress conditions. The inability of TIE2^L914F^ cells to orient in flow could be partially explained by the polarity defect that has previously been shown in HUVEC TIE2^L914F^ model where TIE2 ^L914F^ HUVECs formed enlarged vascular lumen [32]. Interestingly, the TIE2^L914F^ iECs also exhibited significantly larger cell areas compared to TIE2^WT^ iECs under the influence of flow shear stress. The TIE2^WT^ iEC cell area shrunk after addition of flow which is consistent with the known phenomenon wherein WT cells exhibit reduced cell area under flow conditions compared to static environments due to an increased proliferation rate when shear stress is present [60]. Consequently, a larger cell area of TIE2^L914F^ iECs shows an insensitivity to flow induced phenomenon described in WT and could partly explain the dilated vessels observed in VM patients, even without the increase in EC proliferation. Increase in cell length is also observed in electron microscopy analysis of TIE2 mutation positive VM lesions [40]. It should also be noted that we observed no significant differences in cells morphology of iECs without the presence of flow. This differs to our previous studies on HUVECs model, where ECs expressing TIE2^L914F^ exhibited fibroblastic-like cell appearance with cellular extensions. However, general morphology of normal iECs is more “spindle cell-like” than “cobblestone” as in HUVECs hindering the detailed comparison of the effect of mutation on cell morphology between these models. The characteristics iECs morphology could be due to a lack of flow during differentiation that can affect cell shape through natural epigenetic factors that are active in blood vessels.

In EC transplantation assay in SCID mice TIE2^L914F^ iECs formed vessels with increased diameter when compared to TIE2^WT^. The disparity between the TIE2^WT^ and TIE2^L914F^ iEC xenografts was less pronounced than previously observed in a HUVEC^L914F^ model, where HUVECs formed prominent lesions filled with blood in relatively short period after transplantation. This discrepancy could be attributed to several factors. In the HUVEC^L914F^ model, the lesion´s fast growth could be artificially accelerated by an overexpression of TIE2^L914F^, when compared to endogenous levels in TIE2^L914F^ iECs. It remains to be elucidated if extended time would have generated VM-like lesions in TIE2^L914F^ iEC mice. Considering that the TIE2^L914F^ mutation results in overgrowth of vein-like vascular channels, it would also be informative to replicate the experiments using a differentiation protocol that guides iPSCs toward venous iECs [61] enabling comparison of the different EC subtype in VM formation. Another limitation of our study is that we generated cells with a homozygous TIE2^L914F^ mutation rather than heterozygous. A single hit in one allele is often sufficient to induce the formation of VMs, and most patients typically present with a heterozygous TIE2^L914F^ mutation [30, 58]. Therefore, further experiments would be necessary to elucidate potential differences between the homozygous and heterozygous states of the TIE2^L914F^ mutation in vitro and their respective implications in the pathogenesis of VMs.

Overall, our findings demonstrate the suitability of developing iPSC models for studying vascular anomalies. By analyzing the mutation's influence on the cellular and molecular levels, we have laid the foundation for a deeper understanding of the mechanisms underlying VMs. This innovative approach provides a robust platform for future investigations into the intricacies of these anomalies, potentially leading to significant advancements in therapeutic interventions and targeted treatments.

## Materials and methods

*iPSC culture* OdinCas9-hiPSCs [33] were maintained in the Cellartis DEF-CS 500 Culture System (Takara Bio), according to manufacturer’s instructions. All cell lines were cultured at 37 °C with 5% CO_2_. Cell lines were authenticated by STR profiling and tested negative for mycoplasma. Cells were adapted to mTESR system (STEMCELL Technologies) prior to differentiation. iPSCs cultured in DEF were dissociated into single cells with TrypLE Select (Thermo Fisher Scientific, 12,563–029) and plated on Matrigel CLS354277 (Corning) at a density of 35,000 cells/cm^2^ in 50% mTeSR1 medium with 10 mM Y27632 and 50% DEF medium with GF3. After 24 h, the medium was changed to 75% mTeSR1 and 25% DEF, the same ratio was used for media change after 48h. 72 h later, cells were passaged again on Matrigel CLS354277 to 100% mTeSR system. They were cultured in mTeSR for at least 2 more passages before they were used for differentiations. Brightfield photos of cell culture were taken with the Incucyte S3 (Sartorius).

*Cell line generation* Thirty-six hours prior to transfections, 40 k/cm^2^ OdinCas9-hiPS [33] cells were seeded into 6-well plates. Sixteen hours prior, Cas9 was induced with 10 µg/ml of doxycycline for 1 h. 2–3 h before transfection medium was changed to increase the likely-hood of timing a proliferative log phase. The iPSCs cells were transfected with Fugene-HD reagent using a 3.5:1 transfection reagent to DNA ratio and a 3:2 ratio of sgRNA: template; reverse transfection protocol was used. For transfections, 40 k/cm^2^ cells were seeded in 96-well format directly onto prepared transfection complexes with 60ng of DNA per well. Biorender.com was used for a schematic presentation of cell line generation in Fig. 1A. Sequences used in cell line generation are available in supplementary file named 'Supplementary_Sequences.xlsx'.

*DT treatments in vitro* Transfected iPSCs were treated with 20 ng/mL DT from day 3 after transfections. DT-supplemented growth medium was exchanged daily until negative control cells died.

*Karyotyping* Cells were thawed in DEF system and passaged to a T25 flask where they were cultured in mTeSR system until they reached 80% confluency. Cells were prepared for karyotyping accordingly to the protocol provided by Cell Guidance Systems and shipped to them for analysis. Karyotyping was evaluated by G-banding.

*Genomic DNA extractions and next-generation Amplicon sequencing* DNA was extracted from cells using the QuickExtract DNA extraction solution (Lucigen) as per the provided manual. Subsequently, amplicons of interest derived from genomic DNA samples were scrutinized on a NextSeq platform (Illumina). In summary, specific genomic sites underwent amplification in the initial round of PCR using the following primers: F_TIE2 (CAGGGCCACTGATGAGTCGAT) and R_TIE2 (TCGGCAGCGAAGTGAAGGAG). The first PCR employed NEBNext Q5 Hot Start HiFi PCR Master Mix (New England Biolabs) in 15 μL reactions, with 0,5 μM of primers, and 1,5 μL of genomic DNA as a template. The PCR protocol comprised an initial denaturation at 98 °C for 30 s, 30 cycles of [98 °C for 10 s, annealing at the temperature calculated for each primer pair's genomic binding regions using NEB Tm Calculator for 20 s, and 72 °C for 20 s], followed by a final extension at 72 °C for 2 min. Post-amplification, PCR products were purified using the HighPre PCR Clean-up System (MagBio Genomics), and their size and DNA concentration were assessed on a Fragment Analyzer (Agilent). A second round of PCR added unique Illumina indexes to the PCR products using KAPA HiFi Hotstart Ready Mix (Roche). Indexing primers were introduced in a 50 μL reaction volume, with 1 ng of purified PCR product from the initial PCR serving as the template. The cycling conditions for this step included an initial extension at 72 °C for 3 min, followed by an initial denaturation at 98 °C for 30 s, then 10 cycles of [98 °C for 10 s, 63 °C for 30 s, and 72 °C for 3 min], capped by a final extension at 72 °C for 5 min. The final PCR products underwent purification using the HighPre PCR Clean-up System (MagBio Genomics) and were analyzed using a Fragment Analyzer (Agilent). Libraries were quantified with a Qubit 4 Fluorometer (Life Technologies), pooled, and subsequently sequenced on a NextSeq instrument (Illumina).

*Amplicon-seq data analysis* Amplicon-seq sequencing data underwent demultiplexing with bcl2fastq software, followed by analysis using a Perl implementation of a previously described Matlab script [62]. For the quantification of base editing frequencies, the sequencing reads were examined for matches to two 10 bp sequences flanking both sides of an intervening window, where indels or base edits might manifest. Reads without matches (with a maximum allowance of 1 bp mismatch on each side) were excluded from the analysis. If the length of the intervening window deviated from the reference sequence, the sequencing read was categorized as an insertion or deletion, accordingly. The frequencies of insertions or deletions were computed as the percentage of reads classified as such within the total analyzed reads. Reads with intervening windows exactly matching the reference sequence were classified as not containing an indel. For these reads, the frequencies of each base at each locus within the intervening window were calculated and utilized as the frequencies of base edits.

*Differentiation to blood vessel ECs.* iPSCs were differentiated to iECs accordingly to previously published modETV2 protocol [36]. Shortly, on day 0 cells were seeded at 55 k/cm^2^ in mTeSR system; day 1: cells were induced to mesoderm with S1 medium (DMEM/F-12 GlutaMAX (Gibco, 10,565,018), 60 µg/ml ascorbic acid (Merck, A8960), 6 µM CHIR99021 (Merck, SML1046)). Day 3: Cells were transfected with modETV2 RNA (Trilink, WOTL86408; sequence in 'Supplementary_Sequences.xlsx') and kept in modETV2 medium (DMEM/F-12 GlutaMAX, 60 µg/ml ascorbic acid, 50 ng/ml VEGF165 (ProSci, PRSI96-773), 50 ng/ml FGF2 (MERCK, SRP4037), 10 µg/ml EGF (MERCK, SRP3027), 10 µM SB41542 (MCE, #HY-10431)) until day 6. After differentiation, iECs were dissociated into single cells and sorted into CD31^+^ and CD31^−^ cells using magnetic beads coated with anti-human CD31 antibodies (Miltenyi Biotec, 130–097-418). The purified CD31^+^ iECs were expanded in culture on 6 well plates coated with Attachment Factor Solution (Cell applications, 123–500). Culture medium for iECs was prepared by adding Endothelial Cell Growth medium 2 (EGM2) kit supplements into basal medium (PromoCell, C22111). For experiments, iPSC EC were dissociated into single cells with TrypLE Select (Thermo Fisher Scientific, 12,563–029) for 10 min at 37 °C. They were resuspended in EGM2 medium supplemented with 2% BSA and centrifuged for 5 min at 200 rpm. After, cells were resuspended in appropriate media for experiment and cell count was done on Vi-CELL BLU (Beckman Coulter). Biorender.com was used for a schematic presentation of differentiation in Fig. 2A. modETV2 RNA sequence used is available in supplementary file named 'Supplementary_Sequences.xlsx'.

*Immunofluorescence* iECs were seeded on a 96 well plate and fixed when they reached confluency with 4% formaldehyde for 15 min, followed by permeabilization with 0,1% Triton X100. The samples were blocked in 10% fetal bovine serum (FBS) for 1 h and incubated overnight at 4 °C with TIE2 (Merck, 05–584, dilution 1/100), VeCadherin (Cell Signaling Technologies, D87F2, dilution 1/400), CD31 (BD Pharmigen, 555,444, dilution 1/200), VWR (Sigma Aldrich, HPA001815, dilution 1/100) antibody in 5% FBS. After washing with PBS, cells were incubated with the donkey anti-mouse 488 secondary antibody (A21202), goat anti-rabbit (A21206) in 1:1000 or Phalloidin 647 Plus (ThermoFisher, A30107) in 1:400 with 5% FBS. Hoechst (Thermo Fisher, H3570) in 1:5000 was used to stain the nucleus of the cells. The stained cells were imaged with Yokogawa CV7000S confocal microscope and analyzed with FIJI [63].

*Bulk RNA-seq* iECs were seeded on 12 wells and starved in 0,5% EGM2 overnight. For cell lysis, cell culture medium was removed and lysis buffer mix (includes Proteinase K) from the RNAdvance Tissue (Beckman Coulter) was added to the cells in a 96-well plate on ice. The cell lysates were thoroughly mixed by pipetting and subsequently incubated at 37 °C for 25 min. RNA extraction was performed according to the manufacturer’s protocol on a Biomek i7 Hybrid robotic workstation (Beckman Coulter). Subsequently, mRNA enrichment and the preparation of sequencing libraries were carried out using the KAPA mRNA HyperPrep Kit (Roche) according to the manufacturer’s instructions, using a Tecan Fluent® liquid handler. Library quality was assessed on a fragment analyzer using the SS NGS fragment kit (1–6000 bp; Agilent). Sequencing was performed on an Illumina NovaSeq6000 platform using a 200 cycles SP Reagent Kit v1.5 (Illumina) at a loading concentration of 1,75 nM.

*RNA-seq data analysis* Read quality was assessed using FastQC (v0.11.9) [64], Qualimap (v2.2.2d) [65] and samtools stats (v1.15) [66]. Quality control (QC) metrics for Qualimap were based on a STAR (v2.7.10a) [67] alignment against the human genome (GRCh38, Ensembl v105). Next, QC metrics were summarized using MultiQC (v1.12) [68]. Sequencing adapters were then trimmed from the remaining libraries using NGmerge (v0.3) [69]. A human transcriptome index consisting of cDNA and ncRNA entries from Ensembl (v105) was generated and reads were mapped to the index using Salmon (v1.7.0) [70]. The bioinformatics workflow was organized using Nextflow workflow management system (v22.04) [71] and Bioconda software management tool [72]. Differential expression analysis, accounting for paired samples, was carried out using edgeR (3.40.0) [73], limma (3.54.0) [74], and dream from the variancePartition package (1.28.0) [75]. P-values were adjusted for multiple testing using the false discovery rate method by Benjamini and Hochberg [76]. GO-term enrichment was performed using the enrichGO function of clusterProfiler (4.6.0) [77]. Read pileup plots were generated using Gviz (1.42.0) [78]. Biorender.com was used for a schematic presentation of pathway in Fig. 3G.

*Proliferation assay* iECs were seeded on a 96 well plates at 35 k/cm^2^ in starvation media (bEGM2 + 0,5% supp), normal EC medium (bEGM2 + supp) or expansion media (mod ETV2 medium [36]. Plate was then imaged every 30 min in Incucyte S3 for 5 days. Medium was changed every second day. Proliferation was calculated from percentage of covered area in time. Experiment was repeated 3 times for each condition. AUC was measured in GraphPadPrism 9.4.0 (GraphPad Software, USA) and statistics was calculated with paired t-test also in GraphPadPrism 9.4.0. Paired samples belonged to the same differentiation batch and were tested on the same day.

*Migration assay* was performed using xCELLigence RTCA DP instrument (Agilent) accordingly to the protocol provided by xCELLigence. Shortly, RTCA CIM plate (Agilent, 5,665,817,001) was coated on both sides with Attachment Factor Solution (Cell Applications, 123–500) for 30 min. The lower chamber of the plate was filled with medium without chemoattractant (0,5% EGM2 supplement) or medium with chemoattractant (10% EGM2 supplement). iPSC-EC were seeded to an upper chamber of a CIM plate at a density of 35 k/cm^2^ in medium without chemoattractant. Cells were moved to the xCELLigence RTCA DP instrument 30 min after seeding and cell impedance was measured every 15 min. Experiment was repeated 3 times. AUC was measured in GraphPad Prism 9.4.0 and statistics was calculated with paired t-test also in GraphPadPrism. Paired samples belonged to the same differentiation batch and were tested on the same day.

*AKT phosphorylation assay* Cells were starved overnight in starvation media (EGM2 + 0,5% supp) and half of the samples were incubated for 1h in 1 µg/ml ANG1 (ReliaTech, 300–048) before cell lysis in MSD TRIS buffer supplemented with phosphatase inhibitors (Phospho(Ser473)/Total Akt Whole Cell Lysate Kit, MSD, K15100D-1.). AKT phosphorylation assay was performed on a 96 well plate from mentioned kit accordingly to manufacturer instructors and measured on MESO SECTOR S 600MM (MSD). Experiment was repeated with 6 biological replicates. Statistics was calculated with paired t-test in GraphPad Prism 9.4.0. Paired samples belonged to the same differentiation batch and were tested on the same day. As suggested in the kit, multiplex assay format formula was used: %phosphoprotein = ((2 × phospho-signal)/(phospho-signal + total signal)) × 100.

*Flow Cytometry* For flow cytometry cells were dissociated into single cells and resuspended in blocking buffer (PBS without calcium and magnesium, 2,5 mM EDTA, 5% FBS) for 15 min, centrifuged and subsequently stained for 45 min at 4 °C with antibodies SSEA-4 V450 (BD, 561,156), Oct3/4 APC (Miltenyi biotech, 130–117-709), Nanog AF488 (BD, 560,791), CD31-BV421 (BD, 564,089), CD144-PE (Thermo Fisher Scientific, 12–1449-80) and respective isotype controls according to manufacturer’s indications. Samples were acquired with a BD LSR Fortessa 4L (BD-biosciences) flow cytometer and analysed with FlowJo 10.8.0 software. Experiment was repeated 3 times. Statistics was calculated with paired t-test in GraphPad Prism 9.4.0. Paired samples belonged to the same differentiation batch and were tested on the same day.

*Shear stress induction* was performed on high-throughput microfluidic platform from AKITA by Finnadvance Ltd., Finland. The microfluidic platform comprised of 32 microchannel units arranged according to the footprint of a standard 96-well plate. Briefly, the microchannels were coated with 100 µg/ml human fibronectin (Corning) diluted in PBS (Gibco) for one hour at 37 °C, followed by a single wash with 1X PBS, and subsequent replacement with cell culture medium. Subsequently, we loaded 40 µl of each cell type into their respective fibronectin-coated microchannels at a concentration of 2*10^6^ cells/ml. After 24 h of static culture at 37 °C, 5% CO_2_, flow was initiated on the plate by placing it on the AKITA Wave rocker platform, tilted at an angle of 30 degrees, and set to a speed of 1 RPM. The cells were cultured for five days under this controlled flow condition, allowing them to fully occupy the microchannels. On the fifth day of the experiment, the cells were fixed using 4% PFA and subjected to immunostaining for imaging on Yokogawa CV7000S or Leica Stellaris. To quantify the morphology and count of cells, CellPose 2.0 [79] was utilized to acquire cell ROIs. For this a custom model was trained with approximately 20% of acquired images followed by batch processing. Outliers were cleaned manually in the CellPose GUI and the cell ROIs were transferred to FIJI [63] for analysis.

*Cell lysis* iECs on 6 cm plates were grown in EGM2 supplemented with manufacturer supplement solution. Cells were washed with ice-cold 1 × PBS twice and incubated with 500 µl ice-cold cell lysis buffer (9,1 mM Na_2_HPO_4_, 1,7 mM NaH_2_PO_4_, pH 7,2, 1% NP-40, 0,25% sodium deoxycholate, 150 mM NaCl, 0,1% SDS, 1 mM EDTA), including protease and phosphatase inhibitors cocktails (1:100, P8340 and P5726, Sigma-Aldrich) for 5 min at + 4 °C on a rocker. Lysates were homogenised with 20G needle and centrifuged for 15 min at 13,000*g*, 4 °C.

*Western blot* Cell lysates were treated with 1% β-mercaptoethanol (Sigma-Aldrich) at 95 °C for 5 min. 30 µl of samples were mixed with 10 µl of 4 × loading buffer (0,25 M Tris_HCL pH 6,8, 8% SDS, 40% glycerol, 135 mM bromophenol blue), loaded on SDS-PAGE and run for 10 min at 100 V and 45 min at 200 V, 4%/ 7,5% polyacrylamide gel in 1 × running buffer (0,05% SDS, 25mM Tris, 920 mM glycine). Proteins were transferred to the nitrocellulose membrane in 1 × transfer buffer (20% vol/vol ethanol, 25 mM Tris, 920 mM glycine) for 1 h and 30 min, 100 V, at 4 °C. Membrane was blocked in 5% milk in PBST (1 × PBS, 0.1% Tween 20) for 1 h, washed twice with PBST and incubated with primary antibodies: TIE2 (Merck Millipore, 05–584); pTIE2 (Tyr412, Cell Signaling Technology, 4111S); AKT (Cell Signaling Technology, 9272S); pAKT (Ser473, Cell Signaling Technology, 4060B); β-Actin (Sigma, A5441) diluted 1:1000 in 1% BSA-PBST at 4 °C on a shaker overnight. After 3 × washing with PBST, staining was followed by applying the secondary antibodies:HRP-conjugated goat anti-mouse IgG (Affinipure, 115–035-003); HRP-conjugated goat anti-rabbit IgG (Affinipure, 111–035-003;) diluted 1:10 000 in 1% BSA-PBST at room temperature on a shaker for 1 h. After 3 × 5 min washing in PBST, the protein bands were visualized with enhanced chemiluminescence (ECL) reagent (Lumi-Light, Roche) and detected by Azure Biosystems (600). Image J software was used to analyse signal densities normalized to β-actin band density. Experiment was repeated 6 times. Statistics was calculated with paired t-test in GraphPad Prism 9.4.0.. Formula used: % phosphoprotein = phospho-signal/total signal × 100.

*Animals* All experiments were conducted in accordance with the guidelines and regulations set forth by the Gothenburg Ethics Committee for Experimental Animals, under license number 4123 issued in 2022. Female SCID Beige mice aged between 6 and 8 weeks (CB17.Cg PrkdcscidLystb-J/Crl) were procured from Charles River (Germany) and allowed a minimum acclimatization period of one week upon arrival. The mice were housed in groups, provided ad libitum access to water and food, and maintained on a 12-h light/12-h dark cycle under controlled environmental conditions of 21 °C temperature and 45–55% humidity.

*Xenograft mouse model* To mitigate the risk of immune responses when introducing human cells into mice, we utilized an immunocompromised SCID mouse strain. On the day of the experiment, TIE2^WT^ or TIE2^L914F^ iECs were detached, counted, and diluted in EGM2 medium to a concentration of 1 × 10^6^ cells, then combined with an equal number of MSCs (Lonza, PT-2501), that were previously cultured in MSCGM™ Mesenchymal Stem Cell Growth Medium (Lonza, PT-3001). The cell mixture was kept chilled on ice until needed. Mice were anesthetized with isoflurane, and their flanks were shaved for subcutaneous injection. Prior to each injection, the cells were mixed with growth factor reduced Matrigel Matrix (Corning 354,230), 40 µl fibrinogen (Merck, 605,190) (diluted per manufacturer's instructions), 1 µg/ml VEGF-A 165 (ReliaTech, 300–036), 1 µg/ml FGF-2 (ReliaTech, 300–003), and 1 U thrombin (Merck, 341,576). A final volume of 450 µl of the cell/Matrigel/growth factor suspension was loaded into a syringe and injected subcutaneously into the animal's flank. All plastic equipment and syringe needles were cooled on ice to prevent Matrigel polymerization. Animals were monitored daily, and xenograft size was assessed regularly. Every second day, the animals were weighed. Xenografts were harvested seven days post-implantation and fixed for subsequent processing for tissue histology and light sheet microscopy.

*Tissue histology and immunofluorescence* The xenografts were evenly divided into two parts of equal size. One half underwent optical clearing followed by light sheet microscopy analysis, while the other half was forwarded to HistoCenter located in Mölndal, Sweden, for embedding, cutting, and H&E staining.

*Optical clearing* After being dissected seven days post-implantation, xenografts were fixed overnight in 4% PFA. Subsequently, the samples underwent a series of steps to achieve optical clearing. Initially, they were washed three times in PBS for five minutes each. The discoloration process involved incubating the xenografts in 50% CUBIC 1 solution; 100% CUBIC-1 comprising 25% Quadrol,Thermo Fisher Scientific, 122,262), 25% urea (Sigma Aldrich, U5378), and 15% Triton X100 (Thermo Fisher Scientific, T8787) in 35 g dH_2_0, at 37 °C with gentle shaking for one to three hours, followed by one to four days in 100% CUBIC 1 solution. This was finalized by overnight incubation in a 3% H_2_O_2_ solution at 4 °C. The subsequent immunolabeling and clearing procedures followed the iDISCO + protocol. The xenografts were washed twice in PTx.2 solution (0,002% Triton X-100 in PBS) for one hour at room temperature, then permeabilized for two days at 37 °C. Following this, they were blocked for another two days in blocking solution at 37 °C, and subsequently incubated for four days with primary antibodies (human CD31,Abcam, ab76533) diluted in PTwH/5% DMSO/3% donkey serum (Jackson Immunoresearch, 017–000-121) at 37 °C. After washing in PTwH (0,002% Tween 20, 0,01 mg/ml heparin (Sigma Aldrich, H-3149) in PBS four to five times over 24 h, the samples were incubated with secondary antibodies (donkey anti-rabbit IgG – Life Technologies, A31573) diluted in PTwH/3% donkey serum for four days at 37 °C. This was followed by another round of washing in PTwH four to five times over 24 h. The clearing process comprised two steps. Firstly, the samples were dehydrated at room temperature in a methanol/H_2_O series (20%, 40%, 60%, 80%, 100%, 100%) with one-hour incubation per solution. Subsequently, they were shaken in 66% DCM/33% methanol for three hours at room temperature, then twice in 100% DCM for 15 min each. Finally, the xenografts were incubated in DBE overnight, with one refreshment of DBE after a few hours.

*Light sheet microscopy* Image stacks of cleared xenografts were captured using the LaVision Biotec Ultramicroscope II equipped with an Olympus MVX10 zoom body, which includes an internal 2 × objective lens and an Andor Zyla 5.5 sCMOS camera. For obtaining an overview of the xenografts, the magnification was adjusted to fill the field-of-view, with a light-sheet thickness set to 3,86 µm and a z-step of either 1,93 or 1,6 µm. The light-sheet width was maintained at 100%, and the frame acquisition time was set to 100 ms, ensuring optimal exposure. The laser power was adjusted to fully utilize the dynamic range of the camera. Illumination was provided by three light-sheets from the left, with the thinnest part of the beam utilized across the entire specimen using the dynamic horizontal focus function, employing half the recommended steps. For acquiring high-resolution images, a 6,3 × magnification (effectively 12,6 × magnification) was utilized. The light-sheet width was reduced to 10%, and chromatic aberration correction was applied. A single laser from the left was employed, while other settings remained unchanged. The resulting 3D image stacks, saved in TIFF format, were processed using ImageJ software, where they were merged into one TIFF file per channel acquisition for subsequent analysis. Visualization and analysis of 3D vessel networks within the xenografts were performed using Imaris software (version 10) by Oxford Industries, utilizing the filament tracer module.

*Imaris filament tracer analysis* LSFM data is produced as individual images in a folder, therefore images were imported into ImageJ and saved one image stack (.TIFF) and subsequently converted into Imaris files using ImarisFileConverter (version 10.0.1). Image stacks were loaded into Imaris (version 10.0.1) and subsequently analyzed. For filament tracer analysis, the autopath loops without soma and spines algorithm was used. Segment seed point diameter min and max were set to 5 and 35 µm, whereas the segment seed point threshold differed per xenograft for optimal results. Machine learning was applied to both seed points and segments. Finally, a max gap length of 5 µm and a Terminal segment filter of 15 µm was applied.

*ImageJ Manual Analysis* For manual analysis, lumenized vessel segment diameters were measured in 4 slices per xenograft determined by a random number generator but spread out over the entire xenograft. Based on CD31 staining morphology, lumenization of vessel segments was determined. To limit differential interpretation what constitutes a lumenized vessel, slices from TIE2^L914F^ and TIE2^WT^ xenografts were measured in an alternating fashion. Long uninterrupted lumenized vessels were measured as multiple segments. The number of lumenized vessels was normalized to the surface area of the measured xenograft segment.

*Statistical analysis* Results are expressed mean ± S.E. In each experiment, biological repeats were performed to ensure consistent responses in experiments. No exclusion of outliers was performed in the data analysis of the present study. Statistical significance was assigned at P < 0,05, and statistical difference levels were assigned as follows in the Figures, *P < 0,05, **P < 0,01, ***P < 0,001, ****P < 0,001. Statistical analysis was performed using t-test in GraphPad Prism 9.4.0 (GraphPad Software Inc, CA, USA). To address batch-to-batch variations, we utilized pairwise t-tests for statistical analysis, except in cases where experimental design precluded their use, in which instances unpaired t-tests were employed. All figures subjected to pairwise t-tests included biological replicates linked to each other on the graphs.

### Supplementary Information

Below is the link to the electronic supplementary material.

Supplementary file1 (XLSX 12 KB)

Supplementary file2 (DOCX 5288 KB)

## References

[1] Abrahimi P, Chang WG, Kluger MS et al (2015) Efficient gene disruption in cultured primary human endothelial cells by CRISPR/Cas9. Circ Res 117:121–128. 10.1161/CIRCRESAHA.117.306290 25940550 10.1161/CIRCRESAHA.117.306290 PMC4490936

[2] Kangas J, Nätynki M, Eklund L (2018) Development of molecular therapies for venous malformations. Basic Clin Pharmacol Toxicol 123:6–19. 10.1111/bcpt.13027 29668117 10.1111/bcpt.13027

[3] Kim C (2015) iPSC technology-Powerful hand for disease modeling and therapeutic screen. BMB Rep 48:256–265. 10.5483/BMBRep.2015.48.5.100 25104399 10.5483/BMBRep.2015.48.5.100 PMC4578564

[4] Takahashi K, Yamanaka S (2006) Induction of pluripotent stem cells from mouse embryonic and adult fibroblast cultures by defined factors. Cell 126:663–676. 10.1016/j.cell.2006.07.024 16904174 10.1016/j.cell.2006.07.024

[5] Takahashi K, Tanabe K, Ohnuki M et al (2007) Induction of pluripotent stem cells from adult human fibroblasts by defined factors. Cell 131:861–872. 10.1016/j.cell.2007.11.019 18035408 10.1016/j.cell.2007.11.019

[6] Shi Y, Inoue H, Wu JC, Yamanaka S (2017) Induced pluripotent stem cell technology: a decade of progress. Nat Rev Drug Discov 16:115–130. 10.1038/nrd.2016.245 27980341 10.1038/nrd.2016.245 PMC6416143

[7] Schlaeger TM, Bartunkova S, Lawitts JA et al (1997) Uniform vascular-endothelial-cell-specific gene expression in both embryonic and adult transgenic mice (transcriptionenhancer). Cell Biology 94:3058–3063. 10.1073/pnas.94.7.3058 10.1073/pnas.94.7.3058 PMC20321 9096345

[8] Schnürch H, Risau W (1993) Expression of TIE-2, a member of a novel family of receptor tyrosine kinases, in the endothelial cell lineage. Development 119:957–968. 10.1242/dev.119.3.957 8187650 10.1242/dev.119.3.957

[9] Dumont DJ, Gradwohl G, Fong G-H et al (1994) Dominant-negative and targeted null mutations in the endothelial receptor tyrosine kinase, TEK, reveal a critical role in vasculogenesis of the embryo. Genes Dev 8:1897–1909. 10.1101/gad.8.16.1897 7958865 10.1101/gad.8.16.1897

[10] Patan S (1998) TIE1 and TIE2 receptor tyrosine kinases inversely regulate embryonic angiogenesis by the mechanism of intussusceptive microvascular growth. Microvasc Res 56:1–21. 10.1006/mvre.1998.2081 9683559 10.1006/mvre.1998.2081

[11] Sato TN, Tozawa Y, Deutsch U et al (1995) Distinct roles of the receptor tyrosine kinases Tie-1 and Tie-2 in blood vessel formation. Nature 376:70–74. 10.1038/376070a0 7596437 10.1038/376070a0

[12] Takakura N, Huang XL, Naruse T et al (1998) Critical role of the TIE2 endothelial cell receptor in the development of definitive hematopoiesis. Immunity 9:677–686. 10.1016/S1074-7613(00)80665-2 9846489 10.1016/S1074-7613(00)80665-2

[13] Karar J, Maity A (2011) PI3K/AKT/mTOR pathway in angiogenesis. Front Mol Neurosci 4:1–8. 10.3389/fnmol.2011.00051 22144946 10.3389/fnmol.2011.00051 PMC3228996

[14] Fiedler U, Krissl T, Koidl S et al (2003) Angiopoietin-1 and angiopoietin-2 share the same binding domains in the Tie-2 receptor involving the first Ig-like loop and the epidermal growth factor-like repeats. J Biol Chem 278:1721–1727. 10.1074/jbc.M208550200 12427764 10.1074/jbc.M208550200

[15] Jones N, Dumont DJ (1998) The Tek/Tie2 receptor signals through a novel Dok-related docking protein, Dok-R. Oncogene 17:1097–1108. 10.1038/sj.onc.1202115 9764820 10.1038/sj.onc.1202115

[16] Elamaa H, Kihlström M, Kapiainen E et al (2018) Angiopoietin-4-dependent venous maturation and fluid drainage in the peripheral retina. Elife 7:1–32. 10.7554/eLife.37776 10.7554/eLife.37776 PMC6239434 30444491

[17] Saharinen P, Eklund L, Alitalo K (2017) Therapeutic targeting of the angiopoietin-TIE pathway. Nat Rev Drug Discov 16:635–661. 10.1038/nrd.2016.278 28529319 10.1038/nrd.2016.278

[18] Valenzuela DM, Griffiths JA, Rojas J et al (1999) Angiopoietins 3 and 4: diverging gene counterparts in mice and humans. Biochem Commun P R Vagelos 96:194–199. 10.1073/pnas.96.5.1904 10.1073/pnas.96.5.1904 PMC26709 10051567

[19] Leppänen VM, Saharinen P, Alitalo K (2017) Structural basis of Tie2 activation and Tie2/Tie1 heterodimerization. Proc Natl Acad Sci U S A 114:4376–4381. 10.1073/pnas.1616166114 28396439 10.1073/pnas.1616166114 PMC5410806

[20] Jo G, Bae J, Hong HJ et al (2021) Structural insights into the clustering and activation of Tie2 receptor mediated by Tie2 agonistic antibody. Nat Commun 12:6287. 10.1038/s41467-021-26620-1 34725372 10.1038/s41467-021-26620-1 PMC8560823

[21] Fukuhara S, Sako K, Minami T et al (2008) Differential function of Tie2 at cell-cell contacts and cell-substratum contacts regulated by angiopoietin-1. Nat Cell Biol 10:513–526. 10.1038/ncb1714 18425120 10.1038/ncb1714

[22] Saharinen P, Eklund L, Miettinen J et al (2008) Angiopoietins assemble distinct Tie2 signalling complexes in endothelial cell-cell and cell-matrix contacts. Nat Cell Biol 10:527–537. 10.1038/ncb1715 18425119 10.1038/ncb1715

[23] Yuan HT, Khankin EV, Karumanchi SA, Parikh SM (2009) Angiopoietin 2 Is a partial agonist/antagonist of Tie2 signaling in the endothelium. Mol Cell Biol 29:2011–2022. 10.1128/mcb.01472-08 19223473 10.1128/mcb.01472-08 PMC2663314

[24] Maisonpierre PC, Suri C, Jones PF et al (1997) Angiopoietin-2, a natural antagonist for Tie2 that disrupts in vivo angiogenesis. Science (1979) 277:55–60. 10.1126/science.277.5322.55 10.1126/science.277.5322.55 9204896

[25] Huang L, TCRP& PK (1995) GRB2 and SH-PTP2: potentially important endothelial signaling molecules downstream of the TEK/TIE2 receptor tyrosine kinase. Oncogene 11:2097–2103 7478529

[26] Kim I, Kim HG, So JN et al (2000) Angiopoietin-1 regulates endothelial cell survival through the phosphatidylinositol 3’-kinase/Akt signal transduction pathway. Circ Res 86:24–29. 10.1161/01.RES.86.1.24 10625301 10.1161/01.RES.86.1.24

[27] Kontos CD, Stauffer TP, Yang W-P et al (1998) Tyrosine 1101 of Tie2 is the major site of association of p85 and is required for activation of phosphatidylinositol 3-kinase and Akt. Mol Cell Biol 18:4131–4140. 10.1128/MCB.18.7.4131 9632797 10.1128/MCB.18.7.4131 PMC108997

[28] Jones N, Master Z, Jones J et al (1999) Identification of Tek/Tie2 binding partners. Binding to a multifunctional docking site mediates cell survival and migration. J Biol Chem 274:30896–30905. 10.1074/jbc.274.43.30896 10521483 10.1074/jbc.274.43.30896

[29] Sack KD, Kellum JA, Parikh SM (2020) The angiopoietin-Tie2 pathway in critical illness. Crit Care Clin 36:201–216. 10.1016/j.ccc.2019.12.003 32172809 10.1016/j.ccc.2019.12.003 PMC8843037

[30] Limaye N, Wouters V, Uebelhoer M et al (2009) Somatic Mutations in the angiopoietin-receptor TIE2 can cause both solitary and multiple sporadic venous malformations. Nat Genet 41:118–124. 10.1038/ng.272.Somatic 19079259 10.1038/ng.272.Somatic PMC2670982

[31] Uebelhoer M, Nätynki M, Kangas J et al (2013) Venous malformation-causative TIE2 mutations mediate an AKT-dependent decrease in PDGFB. Hum Mol Genet 22:3438–3448. 10.1093/hmg/ddt198 23633549 10.1093/hmg/ddt198 PMC3736867

[32] Cai Y, Schrenk S, Goines J et al (2019) Constitutive active mutant TIE2 induces enlarged vascular lumen formation with loss of apico-basal polarity and pericyte recruitment. Sci Rep 9:1–12. 10.1038/s41598-019-48854-2 31451744 10.1038/s41598-019-48854-2 PMC6710257

[33] Lundin A, Porritt MJ, Jaiswal H et al (2020) Development of an ObLiGaRe doxycycline inducible Cas9 system for pre-clinical cancer drug discovery. Nat Commun 11:4903. 10.1038/s41467-020-18548-9 32994412 10.1038/s41467-020-18548-9 PMC7525522

[34] Li S, Akrap N, Cerboni S et al (2021) Universal toxin-based selection for precise genome engineering in human cells. Nat Commun 12:2832. 10.1038/s41467-020-20810-z 33479216 10.1038/s41467-020-20810-z PMC7820243

[35] Panda A, Suvakov M, Mariani J et al (2023) Clonally selected lines after CRISPR/Cas editing are not isogenic. CRISPR J 6:176–182. 10.1101/2022.05.17.492193 37071670 10.1101/2022.05.17.492193 PMC10123805

[36] Wang K, Lin RZ, Hong X et al (2020) Robust differentiation of human pluripotent stem cells into endothelial cells via temporal modulation of ETV2 with modified mRNA. Sci Adv 24:6–30. 10.1126/sciadv.aba7606 10.1126/sciadv.aba7606 PMC7439318 32832668

[37] Boscolo E, Limaye N, Huang L et al (2015) Rapamycin improves TIE2-mutated venous malformation in murine model and human subjects. J Clin Investig 125:3491–3504. 10.1172/JCI76004 26258417 10.1172/JCI76004 PMC4588237

[38] Limaye N, Kangas J, Mendola A et al (2015) Somatic activating PIK3CA mutations cause venous malformation. Am J Hum Genet 97:914–921. 10.1016/j.ajhg.2015.11.011 26637981 10.1016/j.ajhg.2015.11.011 PMC4678782

[39] Soblet J, Kangas J, Nätynki M et al (2017) Blue Rubber Bleb Nevus (BRBN) syndrome is caused by somatic TEK (TIE2) mutations. J Investig Dermatol 137:207–216. 10.1016/j.jid.2016.07.034 27519652 10.1016/j.jid.2016.07.034

[40] Nätynki M, Kangas J, Miinalainen I et al (2015) Common and specific effects of TIE2 mutations causing venous malformations. Hum Mol Genet 24:6374–6389. 10.1093/hmg/ddv349 26319232 10.1093/hmg/ddv349 PMC4614705

[41] Szklarczyk D, Franceschini A, Wyder S et al (2015) STRING v10: Protein-protein interaction networks, integrated over the tree of life. Nucleic Acids Res 43:D447–D452. 10.1093/nar/gku1003 25352553 10.1093/nar/gku1003 PMC4383874

[42] Miyamura Y, Kamei S, Matsuo M et al (2024) FOXO1 stimulates tip cell-enriched gene expression in endothelial cells. iScience. 10.1016/j.isci.2024.109161 38444610 10.1016/j.isci.2024.109161 PMC10914484

[43] Potente M, Urbich C, Sasaki KI et al (2005) Involvement of Foxo transcription factors in angiogenesis and postnatal neovascularization. J Clin Investig 115:2382–2392. 10.1172/JCI23126 16100571 10.1172/JCI23126 PMC1184037

[44] Brunet A, Bonni A, Zigmond MJ, et al (1999) Akt promotes cell survival by phosphorylating and inhibiting a Forkhead transcription factor 10.1016/s0092-8674(00)80595-4 10102273

[45] Safran M, Rosen N, Twik M, et al (2022) The GeneCards Suite. In: Practical guide to life science databases. Springer Nature, pp 27–56

[46] Belinky F, Bahir I, Stelzer G et al (2013) Non-redundant compendium of human ncRNA genes in GeneCards. Bioinformatics 29:255–261. 10.1093/bioinformatics/bts676 23172862 10.1093/bioinformatics/bts676

[47] Jeansson M, Gawlik A, Anderson G et al (2011) Angiopoietin-1 is essential in mouse vasculature during development and in response to injury. J Clin Investig 121:2278–2289. 10.1172/JCI46322 21606590 10.1172/JCI46322 PMC3104773

[48] Kim KH, Nakaoka Y, Augustin HG, Koh GY (2018) Myocardial angiopoietin-1 controls atrial chamber morphogenesis by spatiotemporal degradation of cardiac jelly. Cell Rep 23:2455–2466. 10.1016/j.celrep.2018.04.080 29791855 10.1016/j.celrep.2018.04.080

[49] Guzeloglu-Kayisli O, Amankulor NM, Voorhees J et al (2004) KRIT1/cerebral cavernous malformation 1 protein localizes to vascular endothelium, astrocytes, and pyramidal cells of the adult human cerebral cortex. Neurosurgery 54:943–949. 10.1227/01.NEU.0000114512.59624.A5 15046662 10.1227/01.NEU.0000114512.59624.A5

[50] Kim JD, Kang Y, Kim J et al (2014) Essential role of apelin signaling during lymphatic development in Zebrafish. Arterioscler Thromb Vasc Biol 34:338–345. 10.1161/ATVBAHA.113.302785 24311379 10.1161/ATVBAHA.113.302785 PMC3977740

[51] Couto JA, Ayturk UM, Konczyk DJ et al (2017) A somatic GNA11 mutation is associated with extremity capillary malformation and overgrowth. Angiogenesis 20:303–306. 10.1007/s10456-016-9538-1 28120216 10.1007/s10456-016-9538-1 PMC5511772

[52] Chuang MM, Wu SH, Charng MJ, Wu YJ (2022) A novel BMPR2 variant gene in relation with hereditary pulmonary arterial hypertension combined with pulmonary arteriovenous malformations. Acta Cardiol Sin 38:542–545. 10.6515/ACS.202207_38(4).20220210A 35873121 10.6515/ACS.202207_38(4).20220210A PMC9295029

[53] Cullion K, Ostertag-Hill CA, Pan M et al (2023) Ablation of venous malformations by photothermal therapy with intravenous gold nanoshells. Nano Lett 23:7092–7099. 10.1021/acs.nanolett.3c01945 37498114 10.1021/acs.nanolett.3c01945 PMC10773554

[54] Cong L, Ran FA, Cox D et al (1979) (2013) Multiplex genome engineering using CRISPR/Cas systems. Science 339:819–823. 10.1126/science.1231143 10.1126/science.1231143 PMC3795411 23287718

[55] Gore A, Li Z, Fung HL et al (2011) Somatic coding mutations in human induced pluripotent stem cells. Nature 471:63–67. 10.1038/nature09805 21368825 10.1038/nature09805 PMC3074107

[56] Assou S, Bouckenheimer J, De Vos J (2018) Concise review: Assessing the genome integrity of human induced pluripotent stem cells: What quality control metrics? Stem Cells 36:814–821. 10.1002/stem.2797 29441649 10.1002/stem.2797

[57] Chu M, Li T, Shen B, et al (2016) Angiopoietin receptor Tie2 is required for vein specification and maintenance via regulating COUP-TFII. 10.7554/eLife.21032.001 10.7554/eLife.21032 PMC5218530 28005008

[58] Hirose K, Hori Y, Ozeki M et al (2024) Comprehensive phenotypic and genomic characterization of venous malformations. Hum Pathol. 10.1016/j.humpath.2024.02.004 38367816 10.1016/j.humpath.2024.02.004

[59] Chen GH, Yang JG, Xia HF et al (2022) Endothelial cells induce degradation of ECM through enhanced secretion of MMP14 carried on extracellular vesicles in venous malformation. Cell Tissue Res 389:517–530. 10.1007/s00441-022-03657-2 35786766 10.1007/s00441-022-03657-2

[60] DeStefano JG, Williams A, Wnorowski A et al (2017) Real-time quantification of endothelial response to shear stress and vascular modulators. Integr Biol (United Kingdom) 9:362–374. 10.1039/c7ib00023e 10.1039/c7ib00023e PMC5490251 28345713

[61] Rosa S, Praça C, Pitrez PR et al (2019) Functional characterization of ipsC-derived arterial- and venous-like endothelial cells. Sci Rep. 10.1038/s41598-019-40417-9 30846769 10.1038/s41598-019-40417-9 PMC6405900

[62] Gaudelli NM, Komor AC, Rees HA et al (2017) Programmable base editing of T to G C in genomic DNA without DNA cleavage. Nature 551:464–471. 10.1038/nature24644 29160308 10.1038/nature24644 PMC5726555

[63] Schindelin J, Arganda-Carreras I, Frise E et al (2012) Fiji: An open-source platform for biological-image analysis. Nat Methods 9:676–682. 10.1038/nmeth.2019 22743772 10.1038/nmeth.2019 PMC3855844

[64] Andrews S, Krueger F, Seconds-Pichon A, et al (2015) FastQC. A quality control tool for high throughput sequence data. Babraham bioinformatics. Babraham Institute

[65] Okonechnikov K, Conesa A, García-Alcalde F (2016) Qualimap 2: advanced multi-sample quality control for high-throughput sequencing data. Bioinformatics 32:292–294. 10.1093/bioinformatics/btv566 26428292 10.1093/bioinformatics/btv566 PMC4708105

[66] Li H, Handsaker B, Wysoker A et al (2009) The Sequence Alignment/Map format and SAMtools. Bioinformatics 25:2078–2079. 10.1093/bioinformatics/btp352 19505943 10.1093/bioinformatics/btp352 PMC2723002

[67] Dobin A, Davis CA, Schlesinger F et al (2013) STAR: Ultrafast universal RNA-seq aligner. Bioinformatics 29:15–21. 10.1093/bioinformatics/bts635 23104886 10.1093/bioinformatics/bts635 PMC3530905

[68] Ewels P, Magnusson M, Lundin S, Käller M (2016) MultiQC: summarize analysis results for multiple tools and samples in a single report. Bioinformatics 32:3047–3048. 10.1093/bioinformatics/btw354 27312411 10.1093/bioinformatics/btw354 PMC5039924

[69] Gaspar JM (2018) NGmerge: Merging paired-end reads via novel empirically-derived models of sequencing errors. BMC Bioinform 19:536. 10.1186/s12859-018-2579-2 10.1186/s12859-018-2579-2 PMC6302405 30572828

[70] Patro R, Duggal G, Love MI et al (2017) Salmon provides fast and bias-aware quantification of transcript expression. Nat Methods 14:417–419. 10.1038/nmeth.4197 28263959 10.1038/nmeth.4197 PMC5600148

[71] DI Tommaso P, Chatzou M, Floden EW et al (2017) Nextflow enables reproducible computational workflows. Nat Biotechnol 35:316–319. 10.1038/nbt.3820 28398311 10.1038/nbt.3820

[72] Dale R, Grüning B, Sjödin A et al (2018) Bioconda: sustainable and comprehensive software distribution for the life sciences. Nat Methods 15:475–476. 10.1038/s41592-018-0046-7 29967506 10.1038/s41592-018-0046-7 PMC11070151

[73] Robinson MD, McCarthy DJ, Smyth GK (2009) edgeR: a bioconductor package for differential expression analysis of digital gene expression data. Bioinformatics 26:139–140. 10.1093/bioinformatics/btp616 19910308 10.1093/bioinformatics/btp616 PMC2796818

[74] Ritchie ME, Phipson B, Wu D et al (2015) Limma powers differential expression analyses for RNA-sequencing and microarray studies. Nucleic Acids Res 43:47. 10.1093/nar/gkv007 10.1093/nar/gkv007 PMC4402510 25605792

[75] Hoffman GE, Roussos P (2021) Dream: powerful differential expression analysis for repeated measures designs. Bioinformatics 37:192–201. 10.1093/bioinformatics/btaa687 32730587 10.1093/bioinformatics/btaa687 PMC8055218

[76] Benjamini Y, Hochberg Y (1995) Controlling the false discovery rate—a practical and powerful approach to multiple testing. J R Stat Soc Ser B (Methological) 57:289–300. 10.1111/j.2517-6161.1995.tb02031 10.1111/j.2517-6161.1995.tb02031

[77] Wu T, Hu E, Xu S et al (2021) clusterProfiler 4.0: a universal enrichment tool for interpreting omics data. Innovation 2:100141. 10.1016/j.xinn.2021.100141 34557778 10.1016/j.xinn.2021.100141 PMC8454663

[78] Hahne F, Ivanek R (2016) Visualizing genomic data using Gviz and bioconductor BT—statistical genomics: methods and protocols. Methods Mol Biol 1418:335–351. 10.1007/978-1-4939-3578-9_16 27008022 10.1007/978-1-4939-3578-9_16

[79] Pachitariu M, Stringer C (2022) Cellpose 2.0: how to train your own model. Nat Methods 19:1634–1641. 10.1038/s41592-022-01663-4 36344832 10.1038/s41592-022-01663-4 PMC9718665

