# Correction: Human iPSC and CRISPR targeted gene knock-in strategy for studying the somatic TIE2L914F mutation in endothelial cells

**DOI:** 10.1007/s10456-024-09932-w

**Published:** 2024-06-26

**Authors:** Bojana Lazovic, Hoang-Tuan Nguyen, Mohammadhassan Ansarizadeh, Leif Wigge, Franziska Kohl, Songyuan Li, Miguel Carracedo, Jere Kettunen, Luc Krimpenfort, Ramy Elgendy, Kati Richter, Laknee De Silva, Bilada Bilican, Prateek Singh, Pratik Saxena, Lars Jakobsson, Xuechong Hong, Lauri Eklund, Ryan Hicks

**Affiliations:** 1 BioPharmaceuticals R&D Cell Therapy Department, Research and Early Development, Cardiovascular, Renal, and Metabolism (CVRM), BioPharmaceuticals R&D, AstraZeneca, Gothenburg, Sweden; 2 Translational Genomics, Discovery Sciences, BioPharmaceuticals R&D, AstraZeneca, Gothenburg, Sweden; 3 Oulu Center for Cell-Matrix Research, Biocenter Oulu and Faculty of Biochemistry and Molecular Medicine, University of Oulu, Oulu, Finland; 4 Finnadvance Ltd., Oulu, Finland; 5 Data Sciences and Quantitative Biology, Discovery Sciences, BioPharmaceuticals R&D, AstraZeneca, Gothenburg, Sweden; 6 Bioscience Renal, Research and Early Development, Cardiovascular, Renal and Metabolism (CVRM), BioPharmaceuticals R&D, AstraZeneca, Gothenburg, Sweden; 7 Department of Medical Biochemistry and Biophysics, Karolinska Institutet, Stockholm, Sweden; 8 School of Cardiovascular and Metabolic Medicine & Sciences, King’s College London, London, UK

In the original published article, the author name “Lars Jakobsson” at affiliation “Department of Medical Biochemistry and Biophysics, Karolinska Institutet, Stockholm, Sweden” was missing from the author list. Therefore, it results an update (inclusion of the author name Lars Jakobsson) in Acknowledgements, Author contributions and Funding section.

The correct version of author group, Acknowledgements, Author contributions and Funding section are provided in this correction.

The original article has been corrected.

