# Supplementary material for: Human iPSC and CRISPR targeted gene knock-in strategy for studying the somatic TIE2L914F mutation in endothelial cells
Source: Angiogenesis. 2024 May 21;27(3):523–42. doi: 10.1007/s10456-024-09925-9 (PMC11303492; doi:10.1007/s10456-024-09925-9)
Supplement: Supplementary file 2 — Supplementary file2 (DOCX 5288 KB) [file 10456_2024_9925_MOESM2_ESM.docx]

**Material and methods, Supplement figures.**

**FOXO1 localisation:** iECs were seeded on a pre-coated (Attachment Factor Solution (Cell applications, catalog no: 123-500) 96 well plates and starved overnight in starvation media (EGM2 + 0,5% EGM2 supp) when they reached confluency. They were fixed with 4% paraformaldehyde (PFA) for 15 min, followed by a PBS wash. The fixated cells were washed in PBS and incubated in blocking and permeabilization buffer; 10% FBS, 0,1% Triton X , PBS (all from Invitrogen), for 15 min at room temperature. The samples incubated overnight at 4 °C with FOXO1 (Cell Signaling Technologies, #14952, dilution 1/100) antibody in 5% FBS. After washing with PBS, cells were incubated with the donkey anti-mouse 488 secondary antibody (A21202, dilution 1:1000) with 5% FBS. Hoechst (Thermo Fisher, H3570) in 1:5000 was used to stain the nucleus of the cells. The stained cells were imaged with Yokogawa CV7000S confocal microscope, 40x magnification, and analyzed with Columbus 2.9.1.699 (Perkin Elmer Inc.).

**Subcellular localization of total TIE2 and pTie2:** The iECs and HUVECs were seeded at density of 40,000 or 20,000 cells on pre-coated coverslips with Attachment Factor Solution (Cell applications, catalog no: 123-500) in 24 well plate, respectively. After 3 days of culture in that media, the samples were stimulated for 1h with 0,5µg/ml ANG1 (R&D Systems. 923-AN-025) or left un-stimulated Cells were fixed with 4% PFA/1xPBS for 15 min. Following fixation, samples were washed 3x with 0,5% TritonX/1xPBS (PBST) and blocked in 3% BSA-PBST overnight at +4°C. After a PBST wash, samples were incubated overnight at 4°C with TIE2 (Ab33,Merck Millipore 05-584, dilution 1:100) and phospho-Tie2 (Tyr992) (Cell Signaling Technologies, #4221, dilution 1:100) antibodies. After washing with 1xPBS, cells were incubated with Goat anti-mouse Alexa Fluor® 488 (Jackson Immuno Research, 115-545-003) and Goat anti-rabbit Cy™3 (#Jackson Immuno Research, 111-165-003) secondary antibodies (dilution 1:300) for 2 hours at room temperature. DAPI (Sigma-Aldrich, D9542, dilution 1:500) was used to stain the nucleus. Coverslips were mounted on glass slides and imaging was performed using a Leica SP8 FALCON laser scanning confocal microscope. The objective used was a HC PL APO 63x/1.40 OIL CS2 DIC (Oil). Samples were imaged with 405nm diode, white light laser with (3 HyD, 1 PMT) detectors. Images were acquired LAS X 3.5.7 software.


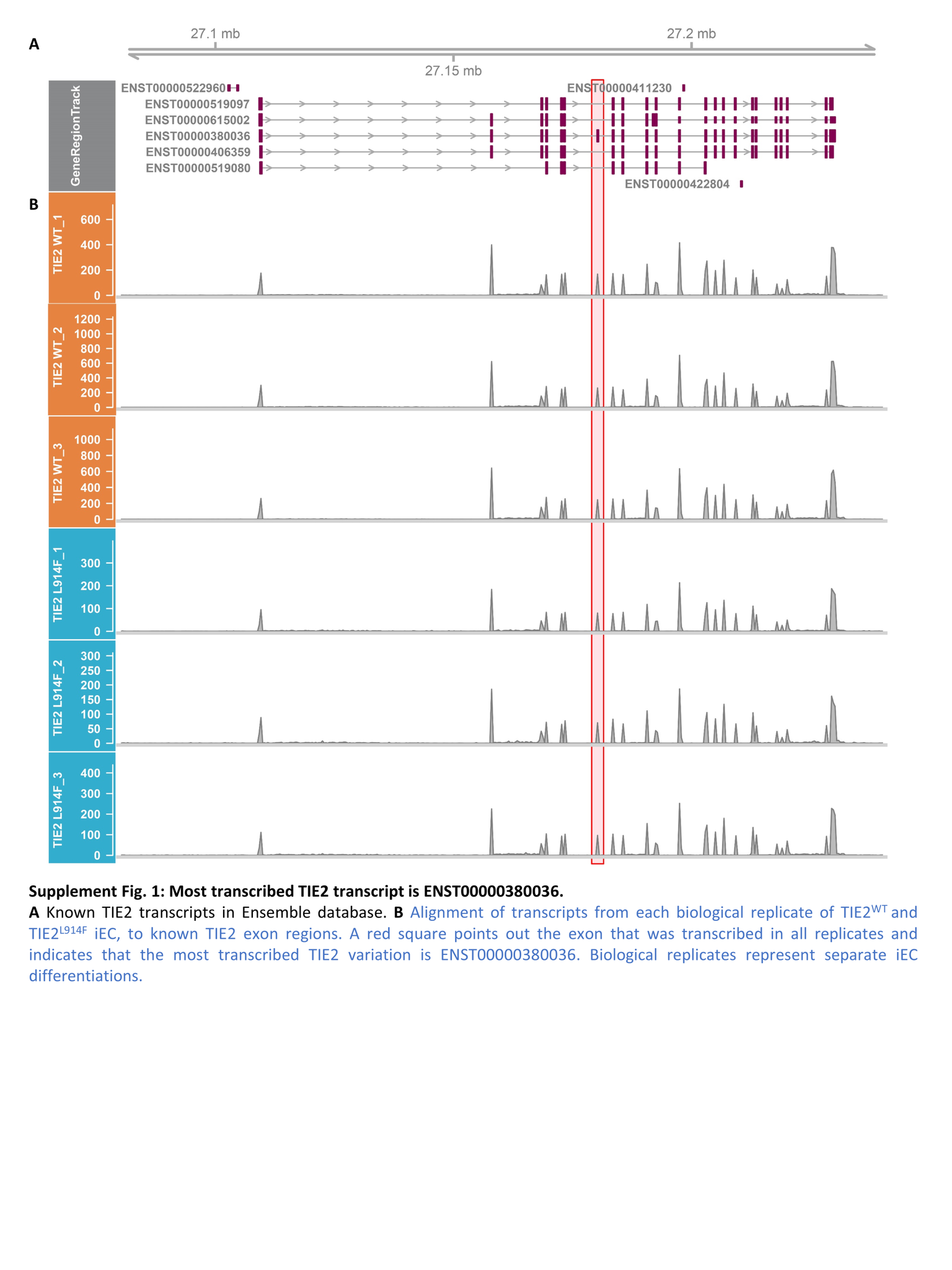


**Supplement Fig. 1: Most transcribed TIE2 transcript is ENST00000380036.**

**A** Known TIE2 transcripts in Ensemble database. **B** Alignment of transcripts from each biological replicate of TIE2^WT^ and TIE2^L914F^ iEC, to known TIE2 exon regions. A red square points out the exon that was transcribed in all replicates and indicates that the most transcribed TIE2 variation is ENST00000380036. Biological replicates represent separate iEC differentiations.


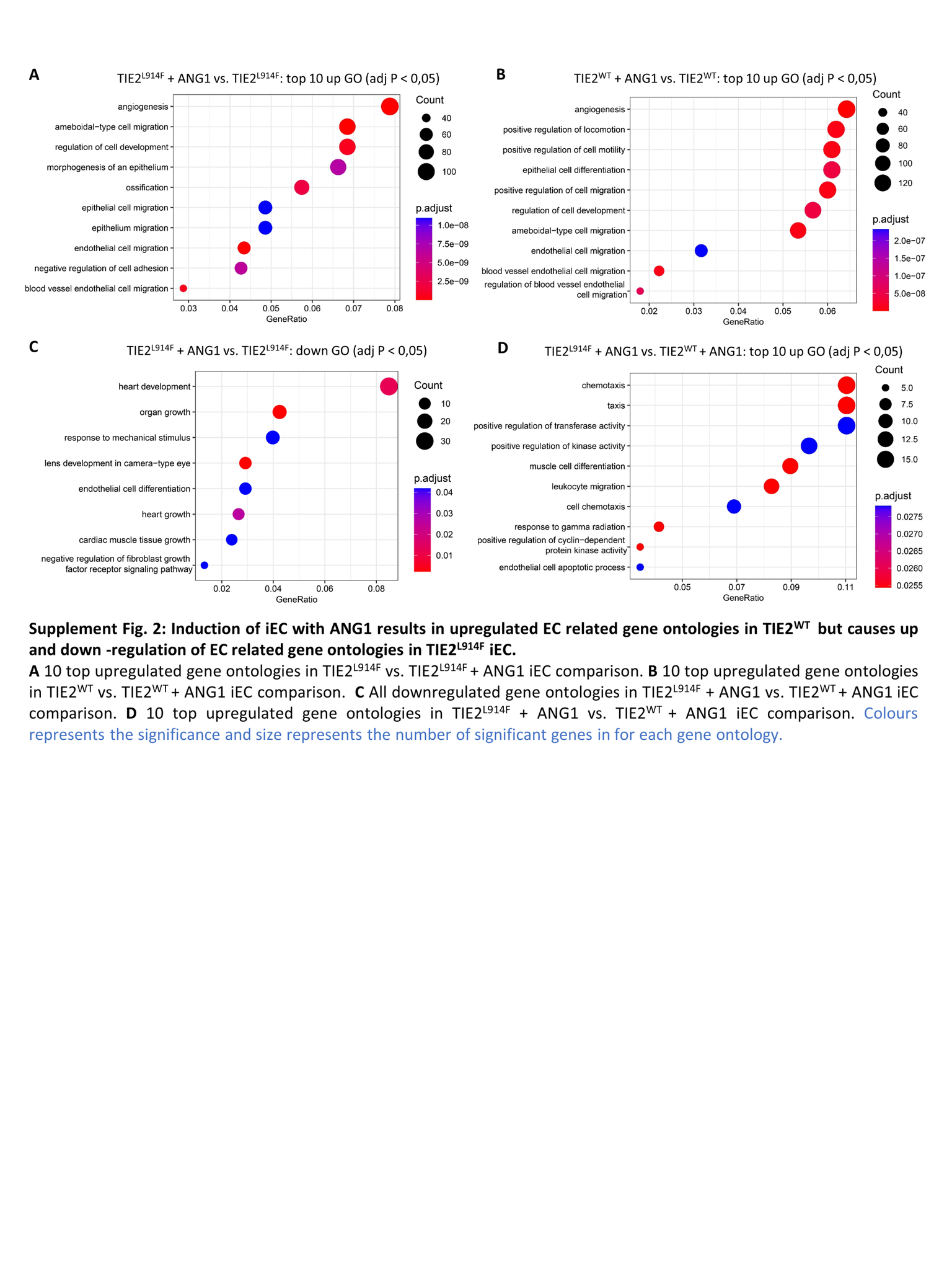


**Supplement Fig. 2: Induction of iEC with ANG1 results in upregulated EC related gene ontologies in TIE2^WT^  but causes up and down -regulation of EC related gene ontologies in TIE2^L914F^ iEC.**

**A** 10 top upregulated gene ontologies in TIE2^L914F^ vs. TIE2^L914F^ + ANG1 iEC comparison. **B** 10 top upregulated gene ontologies in TIE2^WT^ vs. TIE2^WT^ + ANG1 iEC comparison. **C** All downregulated gene ontologies in TIE2^L914F^ + ANG1 vs. TIE2^WT^ + ANG1 iEC comparison. **D** 10 top upregulated gene ontologies in TIE2^L914F^ + ANG1 vs. TIE2^WT^ + ANG1 iEC comparison. Colours represents the significance and size represents the number of significant genes in for each gene ontology.


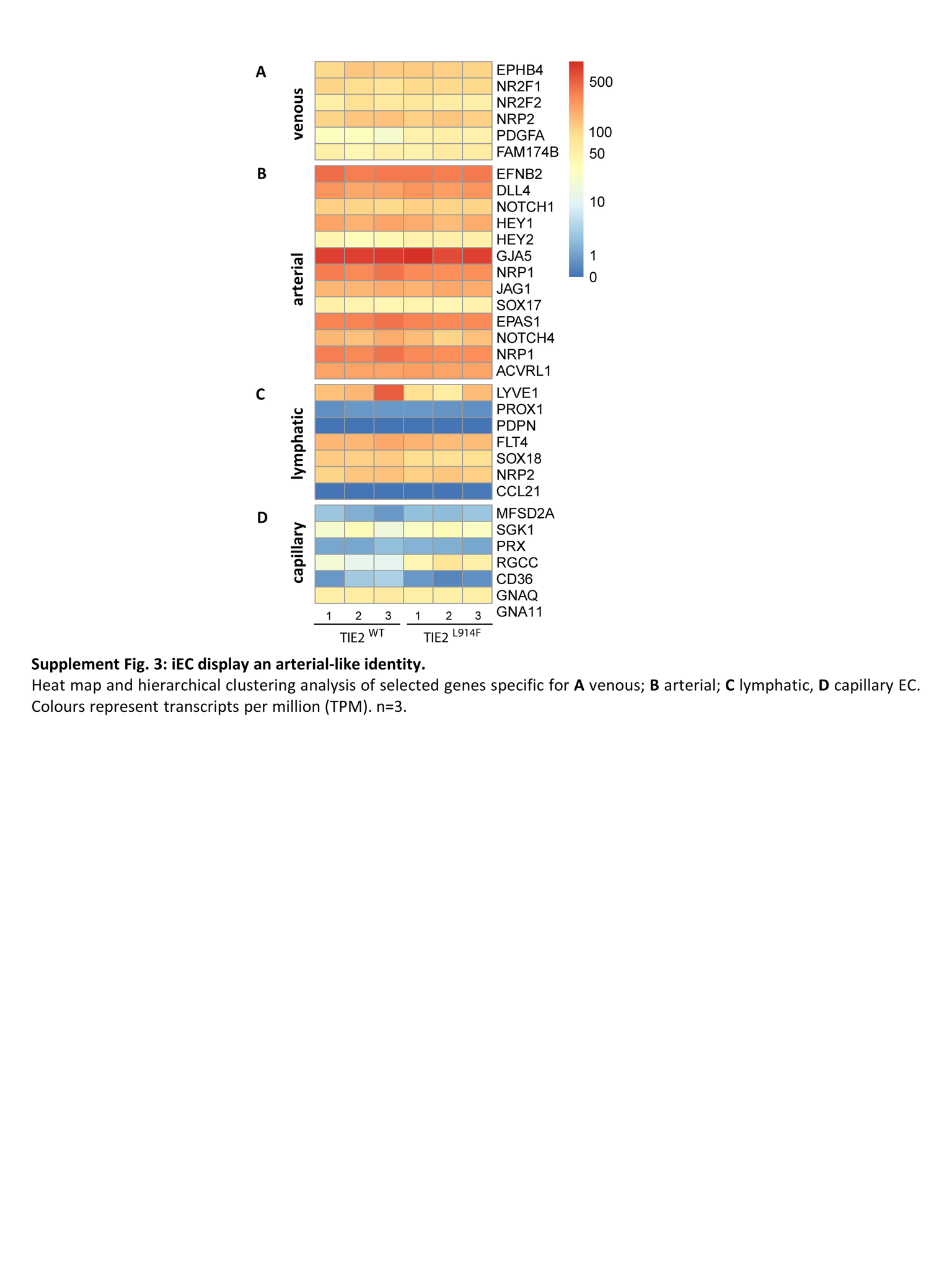


**Supplement Fig. 3: iEC display an arterial-like identity.**

Heat map and hierarchical clustering analysis of selected genes specific for **A** venous; **B** arterial; **C** lymphatic, **D** capillary EC. Colours represent transcripts per million (TPM). n=3.


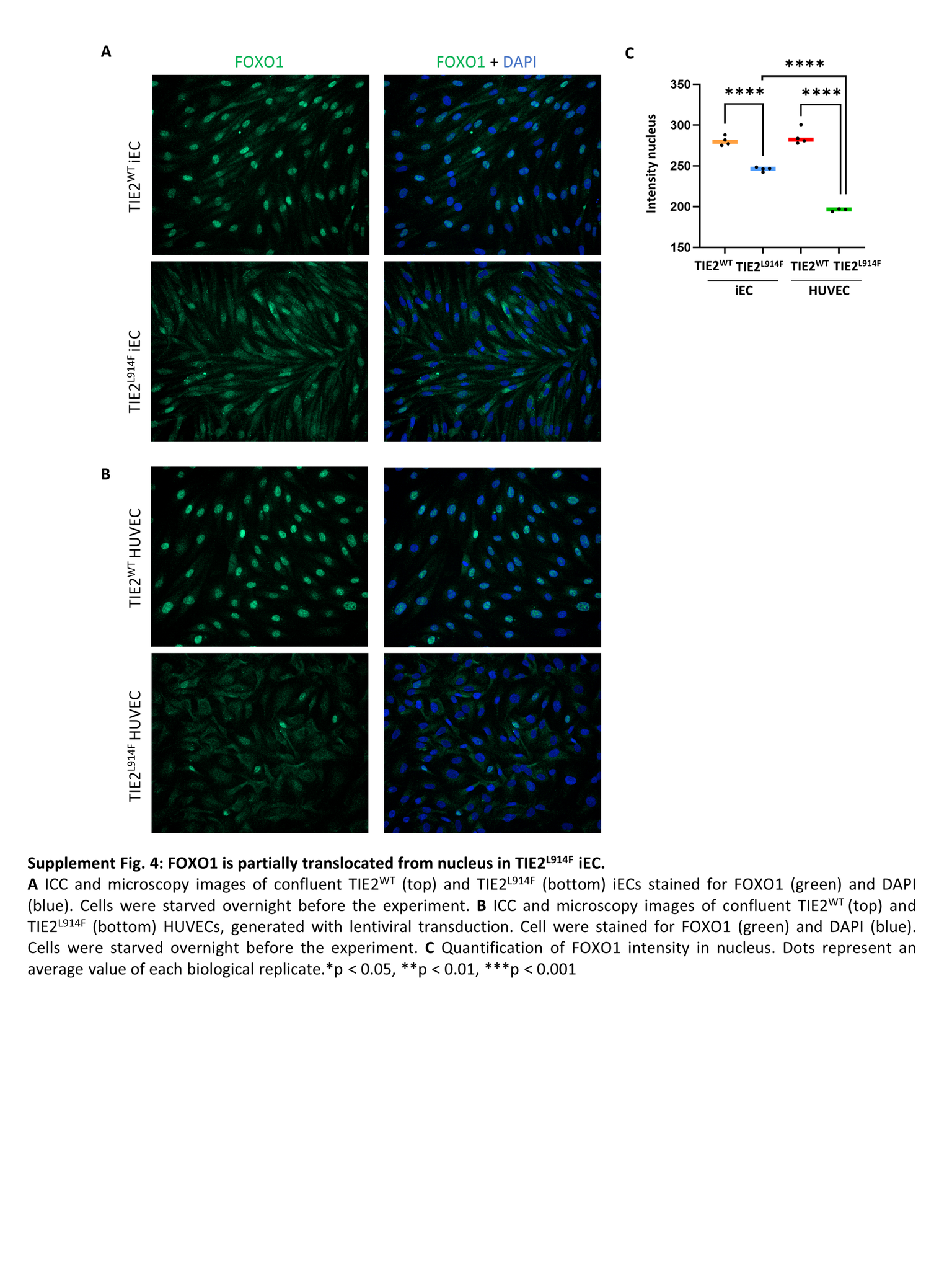


**Supplement Fig. 4: FOXO1 is partially translocated from nucleus in TIE2^L914F^ iEC.**

**A** ICC and microscopy images of confluent TIE2^WT^ (top) and TIE2^L914F^ (bottom) iECs stained for FOXO1 (green) and DAPI (blue). Cells were starved overnight before the experiment. **B** ICC and microscopy images of confluent TIE2^WT^ (top) and TIE2^L914F^ (bottom) HUVECs, generated with lentiviral transduction. Cell were stained for FOXO1 (green) and DAPI (blue). Cells were starved overnight before the experiment. **C** Quantification of FOXO1 intensity in nucleus. Dots represent an average value of each biological replicate.*p < 0.05, **p < 0.01, ***p < 0.001


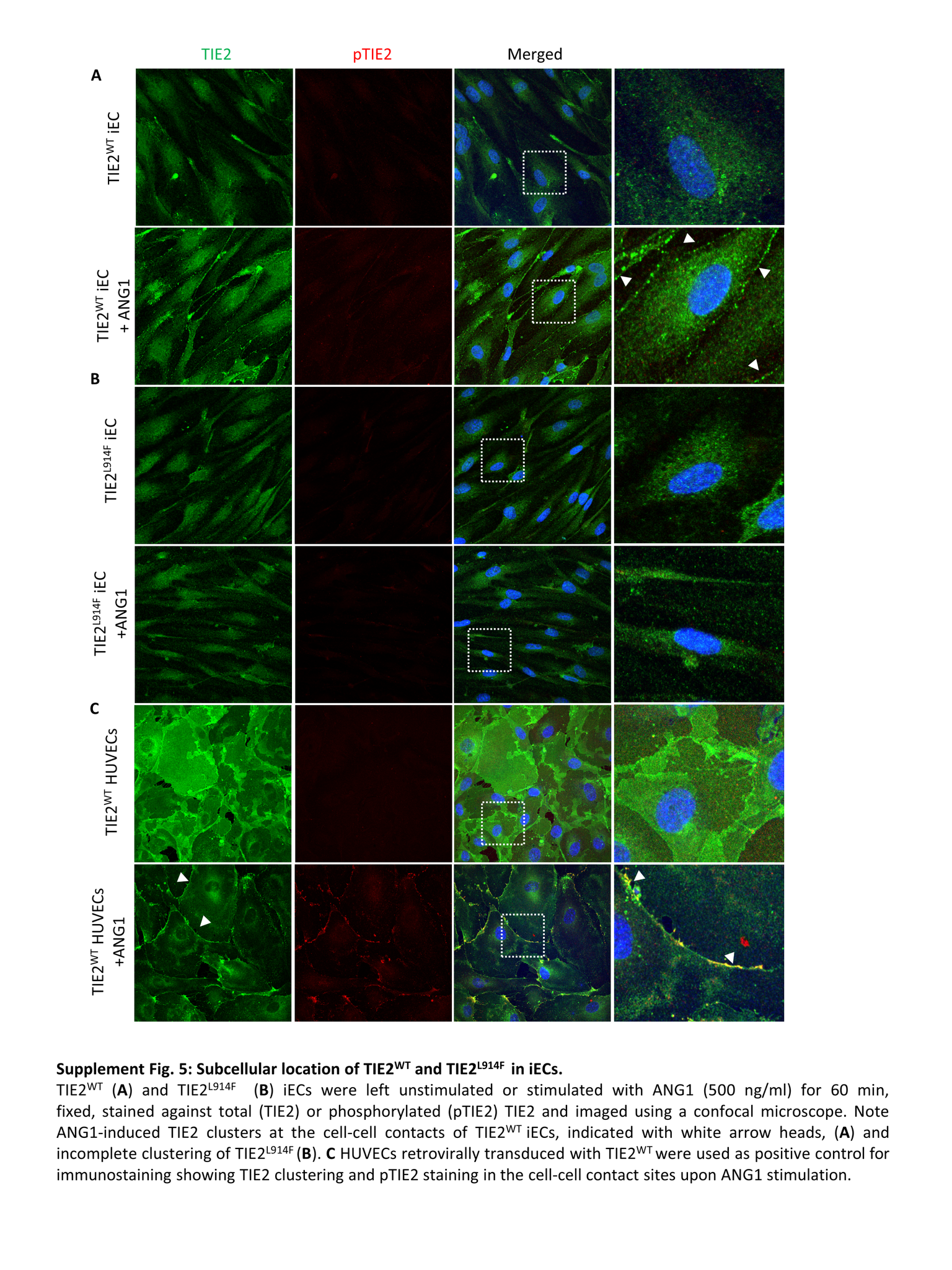
**Supplement Fig. 5: Subcellular location of TIE2^WT^ and TIE2^L914F^ in iECs.**

TIE2^WT^ (**A**) and TIE2^L914F^ (**B**) iECs were left unstimulated or stimulated with ANG1 (500 ng/ml) for 60 min, fixed, stained against total (TIE2) or phosphorylated (pTIE2) TIE2 and imaged using a confocal microscope. Note ANG1-induced TIE2 clusters at the cell-cell contacts of TIE2^WT^ iECs, indicated with white arrow heads, (**A**) and incomplete clustering of TIE2^L914F^ (**B**). **C** HUVECs retrovirally transduced with TIE2^WT^ were used as positive control for immunostaining showing TIE2 clustering and pTIE2 staining in the cell-cell contact sites upon ANG1 stimulation.
